# Organ agar serves as physiologically relevant alternative for *in vivo* bacterial colonization

**DOI:** 10.1128/iai.00355-23

**Published:** 2023-10-18

**Authors:** Melanie M. Pearson, Allyson E. Shea, Sapna Pahil, Sara N. Smith, Valerie S. Forsyth, Harry L. T. Mobley

**Affiliations:** 1 Department of Microbiology and Immunology, University of Michigan, Ann Arbor, Michigan, USA; University of California, Davis, California, USA

**Keywords:** urinary tract infection, *Proteus mirabilis*, animal models, swarming

## Abstract

Animal models for host-microbial interactions have proven valuable, yielding physiologically relevant data that may be otherwise difficult to obtain. Unfortunately, such models are lacking or nonexistent for many microbes. Here, we introduce organ agar, a straightforward method to enable the screening of large mutant libraries while avoiding physiological bottlenecks. We demonstrate that growth defects on organ agar were translatable to bacterial colonization deficiencies in a murine model. Specifically, we present a urinary tract infection agar model to interrogate an ordered library of *Proteus mirabilis* transposon mutants, with accurate prediction of bacterial genes critical for host colonization. Thus, we demonstrate the ability of *ex vivo* organ agar to reproduce *in vivo* deficiencies. Organ agar was also useful for identifying previously unknown links between biosynthetic genes and swarming motility. This work provides a readily adoptable technique that is economical and uses substantially fewer animals. We anticipate this method will be useful for a wide variety of microorganisms, both pathogenic and commensal, in a diverse range of model host species.

## INTRODUCTION

Animal models have a longstanding track record in the study of microbial pathogens, including the fulfillment of Koch’s third postulate [that is, the cultured microorganism should cause disease when introduced into a healthy organism (1)]. Although an excellent *in vivo* model is indispensable for virulence studies, such models may be difficult to establish for fastidious or host-restricted pathogens. Furthermore, even when models exist, complex disease progression can confound experimental interpretation. For example, in the urinary tract, infecting microbes exhibit planktonic growth in urine, adhere or invade the bladder epithelium, ascend into the kidneys, and disseminate into the bloodstream (2, 3). Microbes compete with the host immune response and with established bladder microbiota (4, 5). Each of these challenges can manifest as a chokepoint, or physiological bottleneck, where only a limited number of microbes become established. These bottlenecks have a profound effect on experimental design, particularly in modern large-scale studies, where the number of mutants that can be feasibly studied without random loss is limited due to such founder effects (6 – 9).

Urinary tract infections (UTIs) are one of the most common infections worldwide, affecting most women at least once in their lifetime (10, 11). These infections are even more common in patients with urinary catheters, where a more diverse mix of bacterial species is likely to be the causative agent. In particular, *Proteus mirabilis* is especially problematic in patients with long-term (>30 days) indwelling urinary catheters (12 – 14). This species produces urease, which cleaves urea, abundantly present in urine, into ammonia and carbon dioxide. The resulting pH increase causes precipitation of calcium and magnesium ions naturally present in urine, to form struvite and hydroxyapatite crystals that can block catheter flow and directly damage urinary tract tissue via stone formation (urolithiasis) (15).

Previous studies on *P. mirabilis* virulence have relied on a well-established murine model of UTI (15). Most often, this model involves instillation of bacteria directly into the bladder via a catheter, which can either be removed to study UTI progression in the absence of a foreign body or fully pushed into the bladder to model long-term catheterization (16 – 18). In either case, *P. mirabilis* readily establishes infection and, similar to human UTI, may cause pyelonephritis and urolithiasis (15). In addition to challenging mice with one or two strains at a time, the mouse model has been used to screen pools of mutants from uropathogenic species of bacteria (6, 19).

We aimed to conduct similar large-scale studies to identify genes that contribute to UTI in mice. Toward this goal, we present data showing the experimental bottleneck for the total number of *P. mirabilis* mutants that can be tested in this model without stochastic loss is much narrower than anticipated. We recently built an ordered library containing single transposon insertions in 1,728 genes, representing 45% of predicted genes in *P. mirabilis* strain HI4320 (20). Based on the tight experimental bottleneck, we concluded that screening this library in a murine model would not be feasible. Thus, we devised a method to assay the ordered library on agar plates made by mixing organ homogenates with molten agar to create “organ agar.” In this study, we show that organ agar, as well as agar made from pooled human urine, identified genes that contribute to *P. mirabilis* fitness during experimental UTI in mice. Many of these genes were part of biosynthetic pathways, indicating that organ agar is especially useful for identifying nutritional availability in distinct organ sites. Excitingly, most (six of seven) of the mutants we selected for follow-up study were significantly impaired when cochallenged with the wild-type strain in mice. We anticipate that this economical model will open new avenues of research for many diverse pathogens.

## RESULTS

### The bottleneck effect for *Proteus mirabilis* in a murine model of UTI is much stronger than previously described

**Fig 1:**
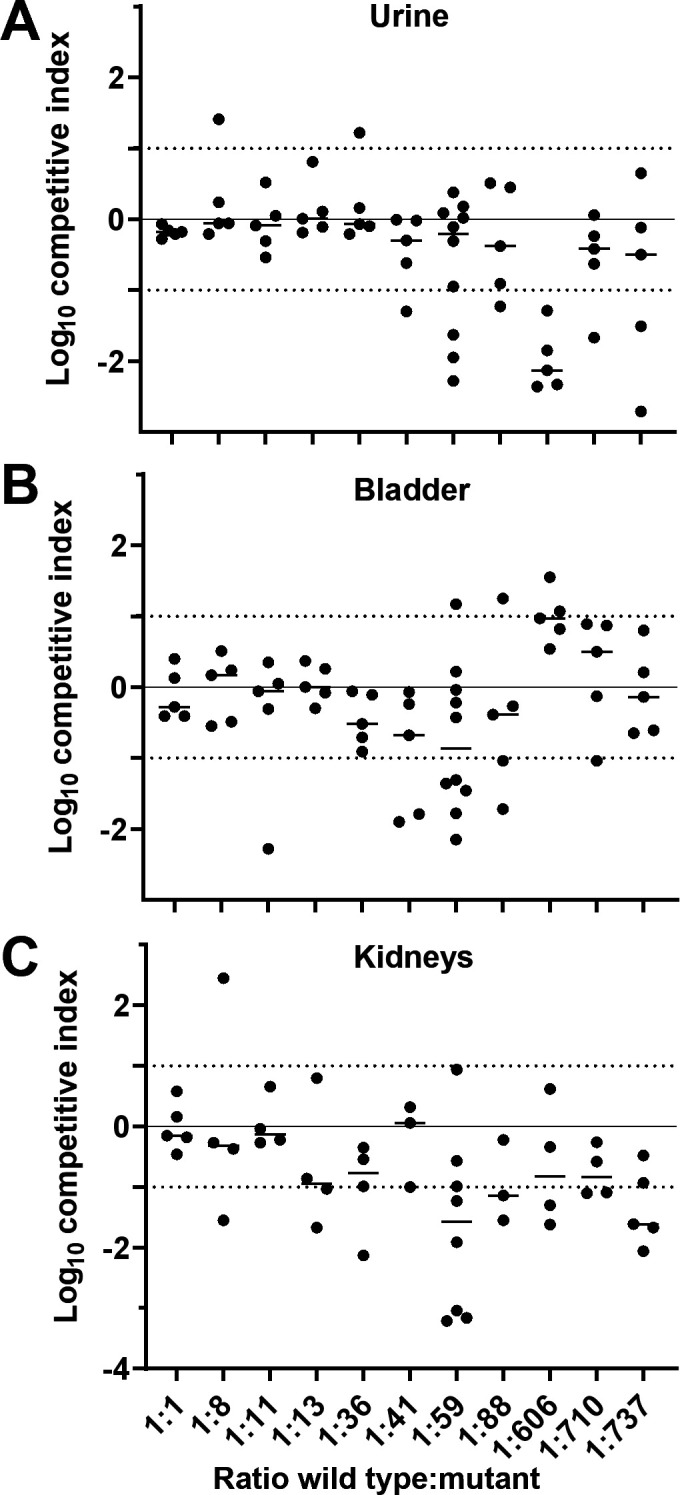
Determining the bottleneck of infection for *P. mirabilis* during UTI. (**A–C**) CBA/J mice were transurethrally inoculated with 10^7^ CFU. The inoculum contained different ratios of wild-type HI4320 and marked mutant *spa47* (kan^R^) that was previously determined to have no fitness defect (23). The measured ratios are indicated on the *x*-axis. At 24 h post-inoculation, (**A**) urine, (**B**) bladder, and (**C**) kidneys were harvested. Each sample was subjected to differential plating to enumerate the ratio of wild type to *spa47* in the organ. A competitive index (CI) was calculated and is plotted on the *y*-axis. Each dot represents a single mouse (*n* = 5–10); bars indicate the median. A log CI of 0 indicates that the wild type and mutant were recovered in the same ratio as were introduced in the inoculum. Dotted lines at ±1 indicate the acceptable maximum variation for this experiment.

Our group and others have successfully enumerated genetic bottleneck effects observed in the traditional model of ascending UTI (6, 21, 22). Although relatively generous infection dynamics of *P. mirabilis* in mice have been estimated for a murine model where a catheter segment is entirely inserted into the bladder lumen (18, 19), we found that the bottleneck for *P. mirabilis* is narrow in the traditional ascending infection UTI model and would, therefore, be problematic for large screening experiments *in vivo* (Fig. 1). To measure the bottleneck, we used a *spa47* mutant that we previously showed has no deleterious effect on bacterial recovery in our mouse model of ascending UTI (23). After testing different input ratios of *spa47* and wild type in cochallenges, we determined that only very small groups of strains can be assembled to avoid stochastic loss of mutants. In urine, up to 100 strains could be tested with the ratio remaining stable throughout the infection, calculated as a log competitive index (CI) of zero (Fig. 1A). However, even at ratios as low as 1:8, we observed 1/5 mice displaying results outside of the ideal range, indicating stochastic loss of the mutant. Bladder samples became unacceptable at an even lower ratio of 1:59, where over half of mice fell outside the preferred range (Fig. 1B). Around 20% of mice with colonization in the urine and bladder had no detectable CFU burden in the kidneys (Table 1). This, in combination with the increased spread of data between biological replicates, caused cochallenge input ratios as low as 1:13 to be problematic (Fig. 1C). Collectively, we demonstrated that the bottleneck during murine UTI with *P. mirabilis* is severe. This is consistent with other studies using uropathogenic *Escherichia coli* (UPEC) (6, 21, 22) although the restriction for *P. mirabilis* is even narrower. For example, in the bladder at 24 h post-inoculation, a ratio of 1:500 exceeded the experimental capacity for testing individual *E. coli* mutants (6) while we found a ratio of 1:59 for *P. mirabilis*. This bottleneck, therefore, limits the feasibility of *in vivo* experimental screens with large pools of mutants. In response, we sought alternative approaches for identifying genes for further study.

**TABLE 1:** Recovery of bacteria from bottleneck experiments

		Number of mice with recovered bacteria		
Target ratio *^a^*	Actual ratio *^b^*	Urine	Bladder	Kidneys
1:1	1:1	5/5	5/5	5/5
1:10	1:8	5/5	5/5	4/5
1:10	1:11	5/5	5/5	4/5
1:25	1:13	5/5	5/5	4/5
1:25	1:36	5/5	5/5	4/5
1:50	1:41	5/5	5/5	3/5
1:50	1:59	10/10	10/10	8/10
1:100	1:88	5/5	5/5	3/5
1:500	1:606	5/5	5/5	4/5
1:1000	1:737	5/5	5/5	5/5
1:1000	1:710	5/5	5/5	4/5

^*a*^ Intended input ratio of *spa47* mutant to wild-type HI4320.

^*b*^ Measured input ratio of *spa47* mutant to wild-type HI4320.

### Development of organ and urine agar

Our goal was to test the fitness of an ordered transposon library built for *P. mirabilis* strain HI4320 (20) in a physiologically relevant condition. Because of the severe bottleneck in mice, which would have required 70 mice to perform a single Tn-seq experiment using pools of 25 mutants from the 1,728-gene-ordered library, we devised a screen that did not require pooling of mutants. For the initial development and screen, we chose outbred male Swiss Webster mice because they are economical. Mouse bladders, kidneys, and spleens were aseptically collected to represent different stages of ascending UTI (cystitis, pyelonephritis, and bacteremia/urosepsis, respectively). Organs were homogenized in water to prevent salt-induced swarming motility by *P. mirabilis* (24, 25). These homogenates were either mixed 1:1 with autoclaved agar or further diluted before mixing with agar. All organs sustained growth of *P. mirabilis*; however, growth on spleens yielded tiny colonies at 24 h and colonies on bladder agar required at least 36 h of incubation to be readily visible. In contrast, the larger and denser kidneys could be diluted up to 1:10 and still sustain growth (Fig. S1). Uninoculated plates showed no outgrowth of microbiota during the duration of the experiment. We selected this 1:10 dilution for screening the ordered library on agar made from kidneys and switched spleens for larger livers to represent disseminated infection, which allowed us to screen 1,728 single gene insertions using organs pooled from only 15 mice.

Similarly, agar made from pooled human urine was adapted for *P. mirabilis* using two modifications. First, the agar was buffered to counteract growth-inhibitory increases in pH due to ammonia released from urease activity (Fig. S2A). Second, the agar concentration was increased to reduce swarming motility. This did not completely eliminate swarming but was sufficient for genetic screening (Fig. S2B). Interestingly, we observed swarming-deficient mutants in the transposon screen, suggesting that this modified urine agar could also be a useful tool for studying swarming motility (Fig. S3).

### Screening of *P. mirabilis* ordered library on organ and urine agar

**Fig 2:**
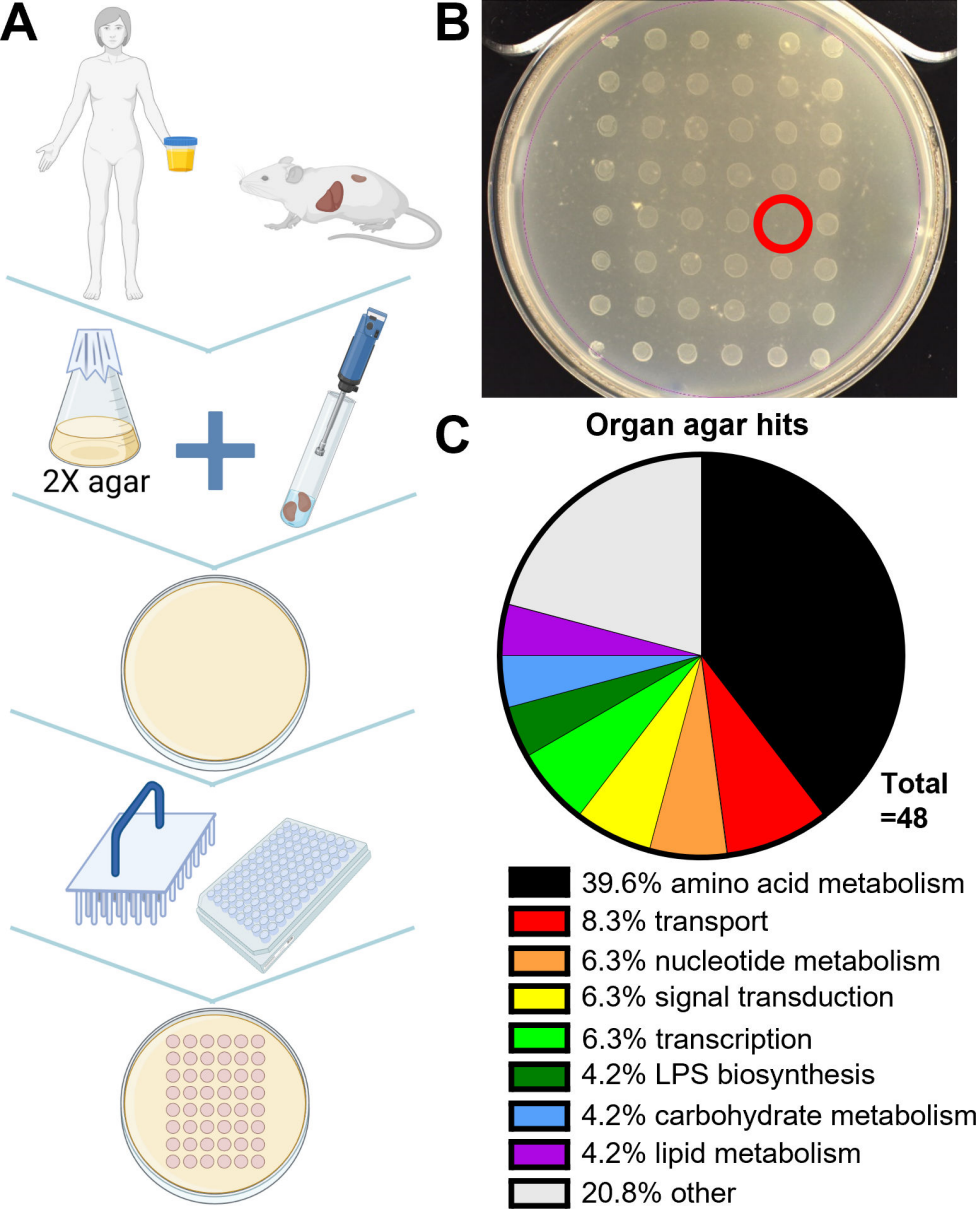
Organ agar screen. (**A**) Schematic for generating organ agar and library screen. (**B**) 1:10 kidney agar with 48 stamped library mutants. One transposon hit (lack of growth) is circled in red. (**C**) Classification of organ agar hits obtained on one or more agars. Genes classified as “other” include singly-binned genes with predicted functions and hypothetical genes.

Mutants in the ordered library were stamped onto liver, kidney, and urine plates and incubated at 37°C, and growth was recorded after 24 and 48 h (Fig. 2; Fig. S3). A total of 48 mutants were qualitatively identified that had growth defects on one or more organ agars (Table 2). The largest category of hits was biosynthetic genes, particularly for amino acids (Fig. 2C). We found genes known to contribute to *P. mirabilis* fitness during UTI (i.e., high-affinity zinc uptake genes *znuA* and *znuC*) (26), as well as pathways known to be important (e.g., TCA cycle) (27). We also identified known contributors to UPEC-mediated UTI (e.g., purines and branched-chain amino acid biosynthesis) (6).

**TABLE 2:** Organ agar hits

Gene	Name	New locus tag	Plate *^a^*	Well *^a^*	OD_600_	Organ *^b^*	Function	General function	PCR confirm *^c^*
PMI0003	*thrC*	PMI_RS00015	216	G4	0.862	U	Threonine synthase	Amino acid metabolism	n/a
PMI0005		PMI_RS00025	213	F6	0.880	U	Conserved hypothetical protein		n/a
PMI0006	*talB*	PMI_RS00030	213	G2	0.818	UK	Transaldolase B	Carbohydrate metabolism	N
PMI0083	*nusB*	PMI_RS00400	210	C11	0.333	K	N utilization substance protein B	Transcription machinery	n/a
PMI0200	*dksA*	PMI_RS00965	211	F9	0.877	K	DnaK suppressor protein	rRNA transcription factor	n/a
PMI0205	*hemL*	PMI_RS00985	219	C2	0.500	UK	Glutamate-1-semialdehyde 2,1-aminomutase	Amino acid metabolism	Y
PMI0206	*erpA*	PMI_RS00990	211	E9	0.576	UK	Putative iron-sulfur protein		Y
PMI0335	*proC*	PMI_RS01600	202	G2	0.644	U	Pyrroline-5-carboxylate reductase	Amino acid metabolism	n/a
PMI0370	*proA*	PMI_RS01770	208	E11	0.727	U	Gamma-glutamyl phosphate reductase	Amino acid metabolism	n/a
**PMI0570 *^d^***	***sucB***	**PMI_RS02805**	**207**	**F6**	**0.863**	**UKL**	**Dihydrolipoamide succinyltransferase component of 2-oxoglutarate dehydrogenase complex**	**TCA cycle^*e*^**	**Y**
**PMI0641**	***sanA***	**PMI_RS03160**	**215**	**C10**	**0.854**	**K**	**Putative transport protein**		**Y**
**PMI0711**	***serC***	**PMI_RS03500**	**219**	**C4**	**0.419**	**UK**	**Phosphoserine aminotransferase**	**Amino acid metabolism**	**Y**
PMI0765	*ompF/ nmpC*	PMI_RS03760	218	G6	0.716	UK	Outer membrane porin	Signal transduction	Y
PMI1151	*znuC*	PMI_RS05555	201	G4	0.775	KL	High-affinity zinc uptake system ATP-binding protein	Transport	Y
PMI1152	*znuA*	PMI_RS05560	209	C1	0.990	K	High-affinity zinc uptake system substrate-binding protein	Transport	n/a
PMI1344	*trpD*	PMI_RS06485	204	B8	0.914	K	Anthranilate synthase component (glutamine amidotransferase)	Amino acid metabolism	n/a
PMI1348	*trpA*	PMI_RS06505	204	C4	0.889	UK	Tryptophan synthase alpha chain	Amino acid metabolism	n/a
**PMI1545**	***guaA***	**PMI_RS07520**	**201**	**D12**	**0.807**	**UKL**	**GMP synthase (glutamine-hydrolyzing)**	**Nucleotide metabolism**	**Y**
PMI1562	*purC*	PMI_RS07610	209	G4	0.880	U	Phosphoribosylaminoimidazole-succinocarboxamide synthetase (SAICAR synthetase)	Nucleotide metabolism	n/a
PMI1833	*cysW*	PMI_RS09045	201	A5	0.609	K	Sulfate/thiosulfate ABC transporter, permease protein	Transport	Y
PMI1875	*purL*	PMI_RS09255	201	C11	0.737	U	Phosphoribosylformylglycineamide synthetase	Nucleotide metabolism	n/a
PMI1912		PMI_RS09435	207	F3	0.737	U	FtsK/SpoIIIE-family protein	Chromosome partitioning	n/a
PMI1945	*ireA*	PMI_RS09585	202	A9	0.714	U	Putative TonB-dependent ferric siderophore receptor	Transport	n/a
PMI2085	*leuB*	PMI_RS10270	207	C5	0.946	U	3-Isopropylmalate dehydrogenase	Amino acid metabolism	n/a
PMI2094	*speA*	PMI_RS10315	201	H1	0.571	K	Biosynthetic arginine decarboxylase	Amino acid metabolism	n/a
PMI2288	*dapD*	PMI_RS11300	210	E12	0.746	K	2,3,4,5-Tetrahydropyridine-2-carboxylate *N*-succinyltransferase	Amino acid metabolism	n/a
PMI2309	*recB*	PMI_RS11420	219	B3	0.241	UK	Exodeoxyribonuclease V beta chain	DNA repair	n/a
PMI2720		PMI_RS13405	213	D5	0.771	U	BMC domain-containing protein		n/a
PMI2821	*argD*	PMI_RS13935	210	A4	0.720	U	Acetylornithine/succinyldiaminopimelate aminotransferase	Amino acid metabolism	n/a
**PMI2870**		**PMI_RS14185**	**208**	**F4**	**0.575**	**UKL**	**Hypothetical protein**		**Y**
PMI2930	*glpD/ glyD*	PMI_RS14485	210	D8	0.727	K	Aerobic glycerol-3-phosphate dehydrogenase	Lipid metabolism	n/a
PMI3027	*aroB*	PMI_RS14975	219	A3	0.734	U	3-Dehydroquinate synthase	Amino acid metabolism	n/a
PMI3028		PMI_RS14980	213	F2	0.946	U	SPOR domain-containing protein	Peptidoglycan binding	n/a
PMI3175	*waaF/ rfaF*	PMI_RS15700	219	B2	0.402	K	ADP-heptose--LPS heptosyltransferase II	Lipopolysaccharide biosynthesis	n/a
PMI3180	*envC*	PMI_RS15725	211	E2	0.564	K	Putative exported peptidase/murein hydrolase activator EnvC	Cell division	Y
PMI3185	*cysE*	PMI_RS15750	212	G7	0.954	UL	Serine acetyltransferase	Amino acid metabolism	n/a
**PMI3210**	***glpK***	**PMI_RS15875**	**214**	**E2**	**0.958**	**U**	**Glycerol kinase**	**Lipid metabolism**	**Y**
PMI3236	*argE*	PMI_RS16000	209	F6	0.812	U	Acetylornithine deacetylase	Amino acid metabolism	n/a
PMI3301	*ilvE*	PMI_RS16410	207	B4	1.016	U	Branched-chain amino acid aminotransferase	Amino acid metabolism	Y
PMI3302	*ilvD*	PMI_RS16415	207	D2	0.913	U	Dihydroxy-acid dehydratase	Amino acid metabolism	n/a
PMI3316	*wecA/ rfe*	PMI_RS16480	219	D6	0.351	U	Undecaprenyl-phosphate alpha-N-acetylglucosaminyl 1-phosphate transferase	Lipopolysaccharide biosynthesis	Y
PMI3319	*rffD*	PMI_RS16495	219	C5	0.398	K	UDP-*N*-acetyl-d-mannosamine dehydrogenase	Carbohydrate metabolism	n/a
PMI3384	*fklB*	PMI_RS16845	213	E11	0.588	K	FkbP-type 22 kDa peptidyl-prolyl *cis*-*trans* isomerase	Chaperones and folding catalysts	N
PMI3431		PMI_RS17100	213	E9	0.974	U	Two-component system sensor kinase	Signaling and cellular processes	N
**PMI3457**	***argI***	**PMI_RS17230**	**204**	**D1**	**0.911**	**UL**	**Ornithine carbamoyltransferase chain I**	**Amino acid metabolism**	**Y**
PMI3528	*metR*	PMI_RS17535	201	A2	0.728	U	LysR-family transcriptional regulator	Amino acid metabolism	Y
PMI3538	*ubiB*	PMI_RS17585	206	A11	0.939	U	Probable ubiquinone biosynthesis protein	Cofactor metabolism	N
PMIt068		PMI_RS16125	219	C1	0.490	K	tRNA	Transcription machinery	n/a

^*a*^ Location of transposon mutants in the ordered library.

^*b*^ Urine, Kidney, Liver.

^*c*^ The computationally predicted location of the transposon was tested by PCR for 20 mutants.

^*d*^ Seven PCR-confirmed mutants that were selected for follow-up studies are highlighted in bold.

^*e*^ *sucB*, primarily known as a component of the TCA cycle, is also involved in amino acid metabolism; it was included as a metabolic gene for purposes of categorization in Fig. 2.

### Selection of candidates for further study

We selected 20 mutant hits for confirmation of the predicted transposon insertion using PCR. Consistent with our previous randomized testing of this library (20), 16 of 20 mutants were confirmed to have the predicted insertion (Table 2; Fig. S4). We selected seven genes to validate with targeted mutagenesis (Table 2, highlighted in yellow). We chose these specific genes to represent (i) the major functional categories from Fig. 2C (amino acid metabolism and purine biosynthesis) and (ii) a range of mutants with phenotypes on either a single or all three types of agar (urine, kidney, or liver).

### Most organ agar mutants had nutritional deficiencies *in vitro*

**Fig 3:**
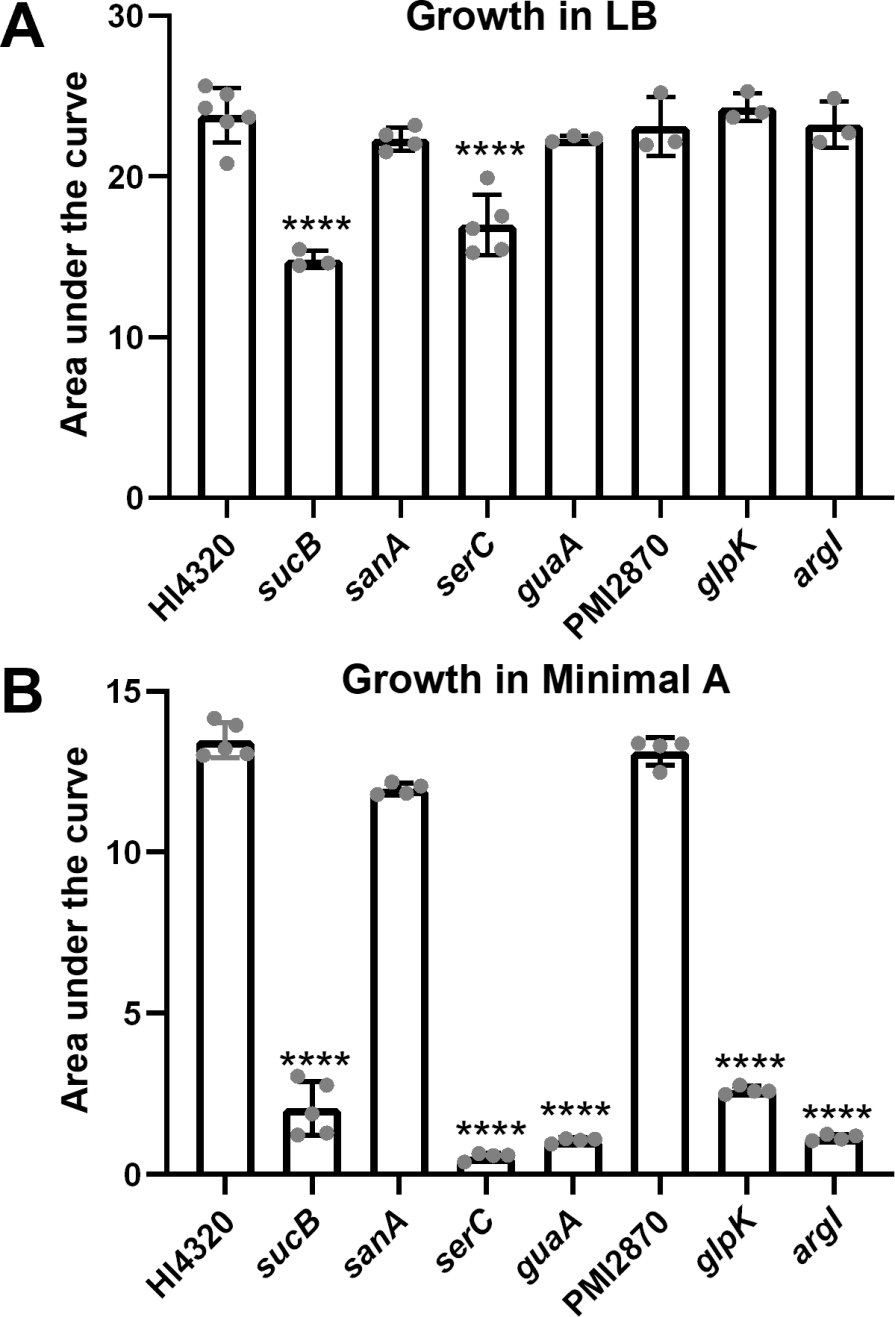
*P. mirabilis* wild type and mutant strains were assayed for growth in (**A**) LB (*n* = 3–6 independent experiments) or (**B**) chemically defined medium Minimal A (*n* = 4–5). Area under the curve was measured after culturing for 20 h in the indicated medium. Most mutants grew well in LB, which was the medium used for obtaining transposon mutants. However, most mutants had a deficiency in Minimal A. Error bars depict SD. Statistical significance was calculated vs wild type using one-way ANOVA with Dunnett’s multiple comparisons test.

**Fig 4:**
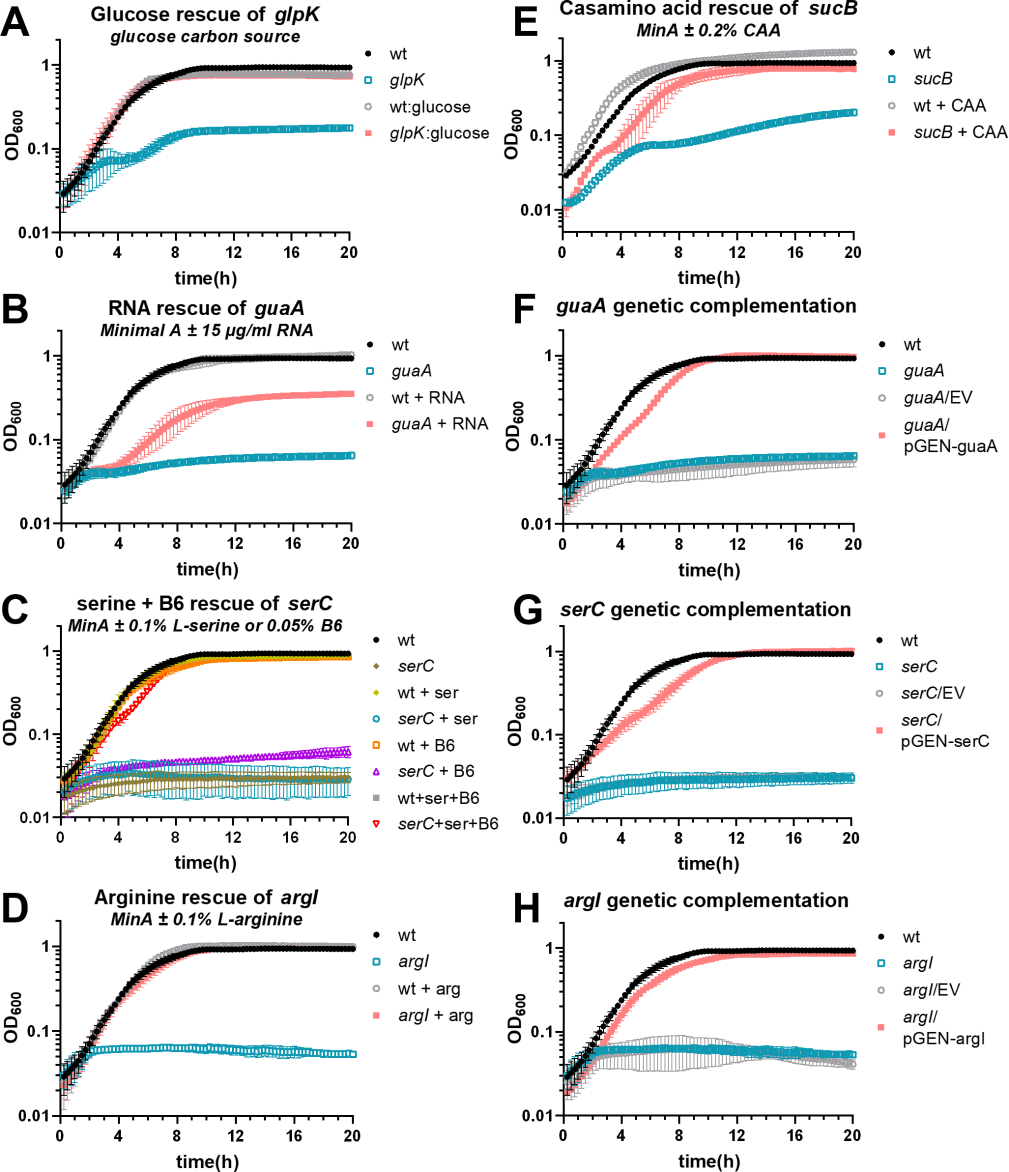
Chemical and genetic complementation is shown for a selection of mutants. For all growth curves, Minimal A medium with 0.2% glycerol as a carbon source was used as the base medium unless otherwise specified. (**A–E**) Chemical complementation growth curves. (**A**) Complementation of *glpK* was achieved by swapping the carbon source to 0.2% glucose. (**F–H**) Genetic complementation growth curves. MinA, Minimal A; B6, pyridoxine HCl; CAA, casamino acids; EV, empty vector (pGEN-MCS). *n* = 3–5 independent experiments for each condition. Error bars show SD.

We examined the growth of all seven of the reconstructed mutants in a variety of media. Most exhibited robust growth in complex LB medium, as expected, because the transposon mutants were originally produced using this medium. The exceptions were *sucB*, which grew at a similar rate as wild type but saturated at a lower density, and *serC*, which grew more slowly but reached the same saturation as wild type (Fig. 3A; Fig. S5A). However, in the chemically defined minimal medium Minimal A, most mutants (5 of 7) had profoundly diminished growth (Fig. 3B; Fig. S4B). Only two, *sanA* and PMI2870, displayed normal growth. Based on the predicted function of each mutated gene, we supplemented Minimal A with appropriate substrates and rescued growth of all mutants. Thus, *glpK* mutant was rescued by swapping the carbon source from glycerol to glucose, *argI* was rescued by the addition of l-arginine, *serC* was rescued with a combination of l-serine and vitamin B6 (28), *guaA* was rescued by the addition of purified *P. mirabilis* RNA, and *sucB* was rescued by the addition of casamino acids (Fig. 4A through E).

We selected three of these mutants for genetic complementation (*argI*, *serC*, and *guaA*). The first two are standalone genes and were cloned as a single fragment with their native promoters. The last gene, *guaA*, follows *guaB* as part of an operon (*guaBA*). This gene was cloned with the *gua* promoter, omitting *guaB*. In all three cases, genetic complementation restored growth in Minimal A (Fig. 4F through H).

### Some mutants had altered swarming motility

**Fig 5:**
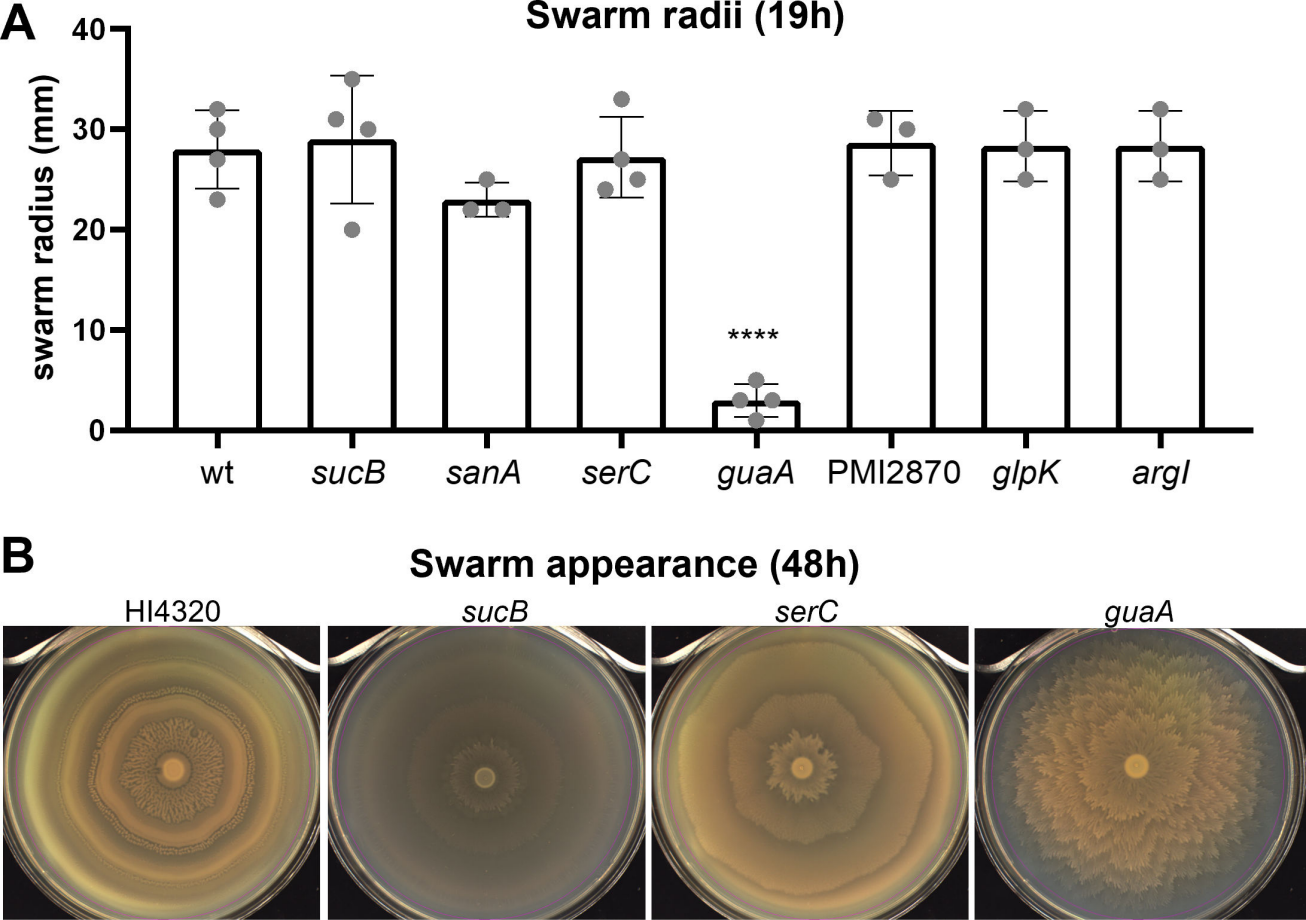
*P. mirabilis* readily swarms in a bullseye pattern on LB agar containing 10 g/L NaCl. (**A**) Quantification of swarm radii after 19 h at 30°C, before wild-type swarms reached the edge of the agar surface. Error bars show SD. Statistical significance was calculated vs wild type using one-way ANOVA with Dunnett’s multiple comparisons test. (**B**) Photos of selected swarms after 48 h incubation at 30°C when swarming and consolidation rings are more visible. Three of 7 of the organ agar hits had strikingly different patterns compared with wild type.

*P. mirabilis* readily colonizes surfaces via swarming motility, including catheters. Because genes involved in swarming often correlate with *in vivo* fitness defects (29, 30), we next assessed swarming motility by these mutants. When assayed for distance swarmed after 19 h of incubation, only the *guaA* mutant had a significant defect (*P* < 0.0001; Fig. 5A). However, *guaA* and two additional mutants, *serC* and *sucB*, exhibited swarming patterns strikingly distinct from the classic wild-type bullseye. These patterns were especially apparent with 48 h incubation time, allowing natural pigmentation to develop (Fig. 5B; Video S1). Swarming by all seven mutants after either 19 or 48 h incubation is shown in Fig. S6.

### Organ agar results were reproducible in another strain of mice

The initial organ agar screen was conducted with transposon mutants using outbred male Swiss Webster mice, selected for low cost and wide availability. We next examined agar made from our standard UTI model strain, female CBA/J mice, with the reconstructed mutants and found comparable results (Table 3). Notably, most phenotypes observed on diluted organs were also seen on agar made from undiluted homogenates, and we genetically complemented the growth defects for *guaA*, *serC*, and *argI* (Fig. S7A). Interestingly, complementation of *argI* led to a hyperaggressive swarming phenotype on urine agar (Fig. S7B), consistent with previous observations that arginine is a swarming cue (31, 32). The major exception was PMI2870, which had a profound defect on all three agar types in the initial screen but no observable defect in the rescreen using CBA/J mice. Because the original screen involved stamping of samples from frozen plates and the rescreen used fresh broth cultures, we reasoned the difference could have resulted from the number of bacteria deposited on the plate. Accordingly, there was a growth defect observed for the PMI2870 mutant on liver agar after 100-fold dilution (Fig. S7C).

**TABLE 3:** Comparison of organ agar and *in vivo* mutant phenotypes

Gene	Name	Organ*^a^*	CBA/J agar*^b^*	In vivo^*c*^	Function
PMI0570	*sucB*	UKL	UKLBS	UBK	Dihydrolipoamide succinyltransferase component of 2-oxoglutarate dehydrogenase complex
PMI0641	*sanA*	K	KS	US	Putative transport protein
PMI0711	*serC*	UK	KS	UBKS	Phosphoserine aminotransferase
PMI1545	*guaA*	UKL	UKLBS	UK	GMP synthase (glutamine-hydrolyzing)
PMI2870		UKL	None^*d*^	UBKS	Hypothetical protein
PMI3210	*glpK*	U	None	US	Glycerol kinase
PMI3457	*argI*	UL	ULB	None	Ornithine carbamoyltransferase chain I

^*a*^ Transposon mutants on agar from Urine (human), Kidney, Liver.

^*b*^ Targetron mutants on agar from Urine (human), Kidney (0.1×), Liver (0.1×), Bladder, Spleen.

^*c*^ Targetron mutants in CBA/J mice from Urine, Kidney, Spleen.

^*d*^ PMI2870 defect was detected in dilution series (Fig. S7C).

Uropathogenic *E. coli* (UPEC) is the most common causative agent of uncomplicated UTI (3). To investigate whether our *P. mirabilis* findings extended to this species, we next tested whether UPEC mutants had similar deficiencies on organ agar. Homologous mutations in UPEC CFT073 were available for five of seven of the target genes. Interestingly, all five of these mutants grew on all organ agars (Fig. S7A). This is consistent with prior work where these mutants had no apparent defect in either human urine *ex vivo* or in murine bladders (6). Notably, UPEC often encodes redundancy that is not seen for *P. mirabilis* (27, 33 – 36).

### Organ agar hits displayed defects in murine ascending UTI model

**Fig 6:**
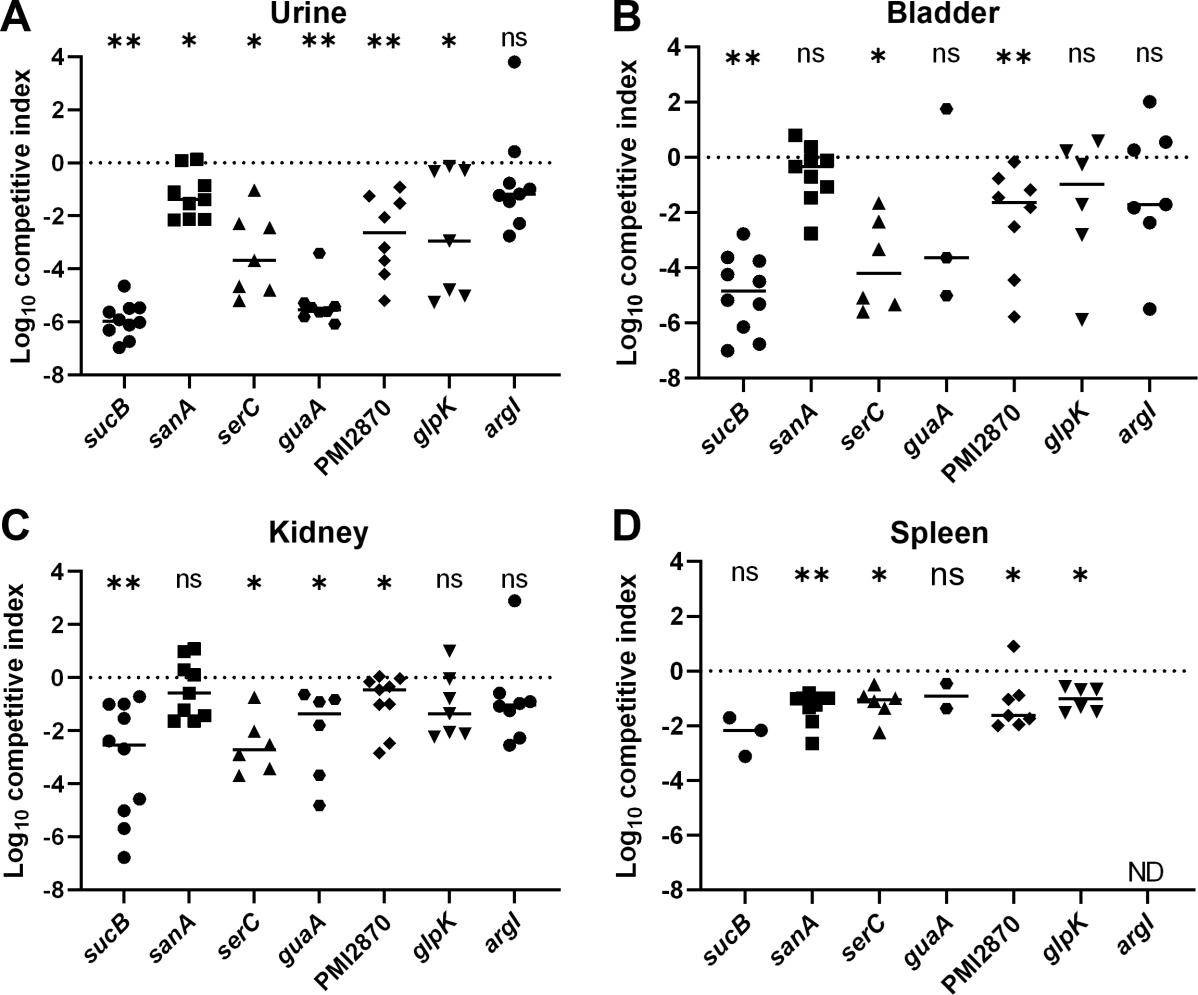
Female CBA/J mice were transurethrally inoculated with a 1:1 mixture of HI4320 (wt) and the indicated mutant. After 7 days, bacteria were quantitatively cultured and competitive indices were calculated. Each symbol represents an individual mouse. Bars indicate median values and the dotted line at *y* = 0 represents no competitive advantage for either wild type or mutant. *P* values were assessed using the Wilcoxon signed rank test (*P < 0.05; **P < 0.01; ns, not significant). ND, not determined; a competitive index could not be determined because no bacteria were recovered.

We were especially interested to see if poor growth of mutants on organ agar translated to deficiencies in establishing UTI. To this end, we transurethrally inoculated bladders of female CBA/J mice with a 1:1 mixture of wild-type *P. mirabilis* HI4320 and each mutant. After 7 days, urine was collected; then mice were sacrificed; and bladders, kidneys, and spleens were removed. In all cases, except *argI*, there was a statistically significant defect in colonization compared with wild type in one or more of the assayed sites (Fig. 6). In three cases (*sucB*, *serC*, and *guaA*), the mutant was almost completely outcompeted by wild type. A comparison of male Swiss Webster murine organ agar with transposon mutants, female CBA/J murine organ agar with targetron mutants, and *in vivo* female CBA/J UTI model fitness is shown in Table 3.

The only mutant that was not statistically significantly outcompeted at any site in murine cochallenge was *argI*, which nevertheless was recovered from urine, bladder, and kidneys by a median of 1–2 log CFU less than wild type. Notably, the overall bacterial burden at 7 days post-inoculation was lower than usual in the *argI* cochallenge (Fig. S8). In summary, lack of growth on organ agar was a highly effective predictor of fitness loss in the urinary tract of mice.

## DISCUSSION

Microbiologists have historically cultured bacteria using media that are reflective of their origins. For example, soil organisms would use soil medium and marine organisms would use seawater (37, 38). Organ-derived media have been used since the inception of bacteriological culture to study mammalian pathogens, sometimes as a method to distinguish related bacterial species (39), but not to our knowledge as a method to screen mutant libraries for host fitness.

*P. mirabilis*, like many other bacterial species, colonizes and infects multiple sites within the human body to cause disease. Specifically, *Proteus* can colonize the gut, skin wounds, and the urinary tract (40, 41). These unique host niches are studied using various *in vivo* models, each with their own limitations (16, 19, 42 – 44). Here, we demonstrated that the urinary tract has strict bottleneck effects in the murine model, requiring the use of many animals for testing mutant libraries. To address this, we present an alternative screening method by generating agar from whole organ samples, greatly reducing the number of mice required for experimentation while increasing feasibility of large screens in models exhibiting limitations.

We screened 1,728 individual *P. mirabilis* transposon mutants from an ordered transposon library, representing 45% of predicted genes in strain HI4320 (20), on human urine and murine kidney and liver agar. Stamping arrayed mutants onto agar only required the use of organs from 15 mice to screen this library. Using a calculated bottleneck of 25 mutants, a comparable Tn-seq experiment would have required 70 mice; thus, organ agar contributes to the conservation of animal lives. In addition to being more ethically and fiscally responsible, this alternative *ex vivo* model strongly correlated with colonization defects *in vivo* and, thus, serves as a proxy for more complex model systems. Indeed, six out of seven mutants had statistically significant defects in the urine, bladder, kidneys, or spleen of mice when examined in the traditional cochallenge model of ascending UTI (Fig. 6) and other validating hits were observed, such as zinc transporter genes *znuA* and *znuC* (26).

Our data show that organ agar is a physiologically relevant medium for testing colonization and virulence factors. However, certain variables may not be modeled as they are *in vivo*. For example, neutrophil recruitment plays an important role in *Proteus* uropathogenesis (45, 46). These non-resident immune cells would likely be in low numbers in the organs of naive mice, and those that are present in organ agar would not exhibit antimicrobial activities such as phagocytosis. Likewise, the architecture of different cell types is disrupted during homogenization. In the uroepithelium, for example, the surface-expressed residues used for bacterial adherence may be heterogeneously distributed throughout the agar. Deeper tissues such as the lamina and muscularis propria, which likely do not directly encounter the bacteria, are exposed in organ agar. Despite these potential pitfalls, organ agar detected fitness factors in functional categories including metabolism, transport, LPS biosynthesis, and even those with unpredicted function.

Most detected hits, 54.3%, fell into a metabolism-related category, suggesting that this method is particularly powerful for identifying the nutritional requirements of microorganisms in their respective host environments. Five of the seven mutants selected for further follow-up analysis were part of well-studied metabolic pathways. Although the lack of growth of most mutants in minimal medium was initially surprising, several mutants exhibiting no growth defects in LB or minimal medium were outcompeted *in vivo*, demonstrating the relevance and sensitivity of *ex vivo* organ agar for discovering new fitness factors beyond metabolism. These findings contrast with a UPEC Tn-seq study, where a much smaller percentage (8%) of detected fitness factors were identified as metabolic (6). Yet, tailored metabolism is increasingly recognized as an integral part of UPEC virulence (47). The *E. coli* accessory genome is remarkably broad (48), and organ agar would greatly facilitate screening a wide variety of UPEC strains for nutritional fitness.

Organ agar also uncovered new connections between nutrition and swarming motility. Swarming is the coordinated, flagella-dependent movement of bacteria across a solid surface. *P. mirabilis* swarming is particularly aggressive compared with other swarm-capable bacterial species and typically manifests as a characteristic bullseye pattern due to successive waves of swarming and consolidation (12). There is a long history establishing the dual importance of biosynthetic and nutrient acquisition pathways to both swarming motility and virulence or colonization fitness, including amino acid biosynthesis, glycolysis, TCA cycle, and peptide import (12). Three of the seven targeted mutants in this study displayed unusual swarm patterns, and all three of these contained mutations in biosynthetic genes (*sucB*, *serC*, and *guaA*). The *guaA* GMP synthase mutant is particularly interesting because of its striking swarming phenotype and a newly identified role for purine biosynthesis in both swarming and colonization of the urinary tract. Previous identification of purines in *P. mirabilis* pathogenesis focused on cyclic AMP, with swarming and virulence defects found for *cyaA* adenylate cyclase and *crp* cyclic AMP receptor protein mutants (34, 49). A different STM study also reported altered swarming for a *serC* mutant although the transposon insertion was upstream of the translational start site and that mutant was able to grow in minimal medium (50). Likewise, serine uptake and biosynthesis have both recently been shown to be important for *P. mirabilis* swarming motility and fitness in a catheterized mouse model of UTI (51, 52). Additionally, when the *argI* mutant was genetically complemented *in trans*, this strain gained an aggressive swarming phenotype, perhaps because extracellular arginine is a potent stimulus of swarming (32). Finally, we note that buffered urine agar mostly constrains *P. mirabilis* swarming but does not eliminate it. This modified urine agar would be a useful tool for screening for swarming-defective mutants. Thus, organ agar may facilitate further studies into the dynamics of swarming motility and its possible contribution to colonization of the urinary tract.

Organ agar reflects distinct niches; for example, the liver and kidney give different results. We speculate that use of particular host genetic backgrounds will also identify unique fitness determinates. For example, knockout mice may change the nutritional landscape for microorganisms. Organ agar could likewise be used to test age or sex differences. Similarly, urine agar could be made using samples from volunteers with specific diets or disease states.

Overall, we demonstrate that *ex vivo* organ agar is a reliable, sensitive method to predict fitness factors in model organisms that can be recapitulated *in vivo*. This is especially important for microorganisms that lack well-developed animal models. Additionally, organ agar can utilize animals that would normally be euthanized to maintain breeding colonies. Using different host models with unique genetic backgrounds, such as knockout mice, may yield surprising and exciting results and mitigate previously mentioned limitations such as immune interactions. We propose that additional refinements of the technique, such as improving plate clarity to allow quantitative densitometry measurements, will further expand the utility of organ agar. We hope that others will use this method to quickly perform large screens of other species and find similar success.

## MATERIALS AND METHODS

### Bacterial strains and plasmids

*P. mirabilis* strain HI4320 was isolated from the urine of an elderly female nursing home patient with a long-term (≥30 days) indwelling catheter (53, 54). This strain is well established as a model organism for *P. mirabilis* virulence studies and readily produces UTIs in mice (15, 16). The *P. mirabilis* HI4320 ordered library contains 1,728 transposon mutants, each insertion within a different open reading frame, and has been described elsewhere in detail (20). Uropathogenic *Escherichia coli* CFT073 was isolated from the blood and urine of a hospitalized patient with acute pyelonephritis and bacteremia (55). *E. coli* homologs of *P. mirabilis* organ agar hits were identified using BLAST, and selected *E. coli* CFT073 transposon mutants were pulled from a previously reported ordered library (6). Bacteria were routinely cultured at 37°C with aeration in lysogeny broth (LB; 10 g/L tryptone, 5 g/L yeast extract, 0.5 g/L NaCl) or on LB solidified with 1.5% agar. All strains and plasmids are listed in Table S1. All strains used in this study can be obtained by contacting the corresponding author.

### Organ and urine agar

For initial development of organ agar, 5 male Swiss Webster mice (outbred, 6- to 7-week-old, Envigo) were humanely euthanized. To facilitate aseptic organ removal, skin and fur were removed from the abdominal region as previously described prior to opening the abdominal cavity (16). Organs (bladders, kidneys, and spleens) were removed, homogenized in 3 mL of water, pooled by organ type, and diluted in a range of ratios in sterile distilled water (Table 4). Three percent of agar was autoclaved, cooled to 55°C, and mixed 1:1 with the diluted organ homogenates. In general, larger organ masses could be diluted further and still support bacterial growth; for example, a 1:10 dilution of kidney homogenate was experimentally determined to sustain reliable growth of wild-type *P. mirabilis* HI4320 (Fig. S1). Because growth on spleen agar was relatively poor, for the subsequent ordered library screen, spleens were swapped for larger livers to represent disseminated infection. Urine agar was made with modifications from a previous protocol (24). Specifically, human urine was collected from seven healthy female volunteers, pooled, and filter sterilized. Aliquots of urine were stored at −20°C. Four percent of agar buffered with 500 mM HEPES, pH 6.8, was autoclaved, cooled to 55°C, and mixed 1:1 with pooled urine. Both organ and urine agar were precisely aliquoted (20 mL) to 100 mm Petri dishes and allowed to solidify and dry at room temperature overnight.

**TABLE 4:** Preparation of organ agar, per plate

Ratio *^a^*	Organs per plate (mL)	Homogenized organ (mL)	Sterile water (mL)	Molten 3% agar (mL)	Total volume (mL)
2× *^b^*	6.7	10	0	10	20
1×	3.3	10	0	10	20
1:2	1.7	5	5	10	20
1:5	0.67	2	8	10	20
1:10	0.33	1	9	10	20

^*a*^ Ratio is determined by dilution of organ before mixing with agar.

^*b*^ For 2× agar, homogenize two organs per 3 mL water; otherwise prepare as for 1× agar.

### Ordered library screen

For the library screen, 15 male Swiss Webster mice were euthanized as above. Livers and kidneys were collected, homogenized, and pooled and then diluted 1:10 with sterile distilled water. Diluted homogenates or pooled human urine were mixed with molten agar and aliquoted to Petri dishes as above. Twenty 96-well plates containing the frozen *P. mirabilis* ordered library were placed on dry ice. A sterile 48-pin stamper was used to transfer bacteria onto organ or urine agar plates, which were then incubated at 37°C for 24 or 48 h. Plates were visually assessed for decreased or absent growth in each spot. Interesting candidates were rescreened to confirm the phenotype. The genetic location of the transposon was confirmed for a selection of 20 of these mutants using PCR with transposon-specific primer CP-7 and a primer flanking the transposon insertion site (Table S2).

### Construction of targeted mutants

Stable insertional mutations of selected genes were generated using the targetron method (23, 56). Briefly, a group II intron fragment was synthesized (eBlocks, Integrated DNA Technologies) to specifically target each gene using the ClosTron prediction algorithm (57). Reprogrammed intron fragments were cloned into pACD4K-CloxP using NEBuilder HiFi DNA Assembly master mix (New England Biolabs) with primers designed to amplify vector or intron templates and confirmed using Sanger DNA sequencing. Targetron-containing plasmids and a source for T7 polymerase, pAR1219 (58), were introduced into *P. mirabilis* HI4320 using electroporation and induced to jump into the specified genes. Insertional mutations in kanamycin-resistant mutants were confirmed using PCR. Targetron plasmids and mutants are listed in Table S1. All primers are listed in Table S2.

### Growth curves and chemical complementation of mutants

Wild type and mutant *P. mirabilis* were cultured separately overnight in LB and then diluted 1:100 into target media for growth curve analysis using a Bioscreen C (Growth Curves USA). Readings were collected at an optical density of 600 nm (OD_600_) every 15 min for 24 h. Each experiment contained three technical replicates and was conducted at least three times. Minimal A is a minimal, chemically defined medium tailored for *P. mirabilis* and was used for most experiments (59). The carbon source in Minimal A was 0.2% glycerol unless otherwise specified. For chemical complementation with RNA, RNA purified from *P. mirabilis* HI4320 or isogenic mutants (RNeasy kit, Qiagen) was pooled together, quantified using a NanoDrop spectrophotometer, and then added to Minimal A at a final concentration of 15 µg/mL.

### Genetic complementation of mutants

Complementation plasmid pGEN-MCS, chosen because it is low copy number and is maintained stably without antibiotic selection, was used for genetic complementation of selected mutants (60). Native *P. mirabilis* promoters were predicted using Softberry (61) and included all predicted DNA-binding protein sites. Coding sequences and promoters were amplified by PCR, cloned using the Gibson method (New England Biolabs), and constructs were confirmed by Sanger sequencing. Complementation plasmids are listed in Table S1, and primers used for cloning are shown in Table S2.

### Swarming assays

Swarming motility was assayed on LB agar containing 10 g/L NaCl as previously described (62). Briefly, strains were cultured overnight in LB (LB 10 g/L NaCl), spotted onto swarm agar, and allowed to dry. Swarm plates were incubated at 30°C. Swarm radii were measured after incubation for 19 h, and swarms were photographed after incubation for 48 h using a Qcount (Advanced Instruments). Swarming experiments were independently repeated at least three times.

### Mouse model of ascending UTI

Bacterial fitness during UTI was assessed using a well-established mouse model (17, 63, 64). Briefly, overnight cultures of *P. mirabilis* were diluted in LB to OD_600_ = 0.194 (~2 × 10^8^ CFU/mL), and then wild type and mutant bacteria were mixed 1:1. Ten female CBA/J mice, aged 5–6 weeks (Jackson Laboratory), were transurethrally inoculated with 50 µL of this 1:1 mixture (10^7^ CFU/mouse). At 7 days post-inoculation, urine was collected; mice were euthanized; and bladders, kidneys, and spleens were harvested. Organs were homogenized and plated to quantify CFU; mutants were distinguished from wild-type colonies using kanamycin. Competitive indices were calculated for each site by comparing the ratio of output wild type and mutant to the ratio of input bacteria (36). Statistical significance of competitive indices was calculated using the Wilcoxon signed rank test.

### Mouse model bottleneck determination

Wild-type HI4320 and a kanamycin-resistant mutant that was previously shown to have no fitness defect in our mouse UTI model, *spa47* (23), were mixed in different ratios to test the limitations of our mouse model. The OD_600_ was recorded to normalize strains prior to mixing and adjusted to target ratios. Aliquots of the input mixtures were taken as samples and plated to determine the actual ratio of mutant:wild type in the inoculum (Fig. 1). The output organ samples collected from CBA/J mice were homogenized and enumerated, via differential plating, to determine mutant and wild-type HI4320 CFU burden. A competitive index calculation was used to demonstrate the output ratio relative to the input ratio (6). A sample that was the same ratio at the beginning and end of the experiment would have log_10_ CI = 0. The acceptable range of error for these experiments was ±1 log (10-fold) CFU.

### Organ agar for targeted mutants

Targetron mutants were tested on organ agar made from female CBA/J mice to directly compare with *in vivo* cochallenges in this standard UTI mouse model. Agar was made as above, but in addition to 1:10 dilutions of liver and kidneys, undiluted “1×” organs were used. For bladder and spleen agar, two organs were homogenized together in 3 mL of water to achieve a 2× concentration of each organ. Bacteria were cultured overnight in LB, adjusted to OD_600_ = 0.1 in LB, aliquoted into a 96-well plate, and stamped onto organ agar. Plates were incubated at 37°C, and growth was photographed at 24 and 48 h. Mutant PMI2870 was additionally screened on urine and 0.1× liver agar in a 1:10 dilution series to determine whether the amount of inoculum affected growth on organ agar.

### Statistics and software

All graphs and charts were plotted and statistics calculated using GraphPad Prism 9. Error bars show SD. To calculate statistical significance for all bar graphs, one-way ANOVA with Dunnett’s multiple comparisons test was used. For mouse cochallenge experiments, statistical significance was calculated using the Wilcoxon signed rank test. *P < 0.05, **P < 0.01, ***P < 0.001, ****P < 0.0001.

## SUPPLEMENTAL MATERIAL

Supplemental Figs S1-S8 iai.00355-23-s0001.pdf Supplemental figures.

Supplemental Tables S1-S2 iai.00355-23-s0002.pdf Bacterial strains, plasmids, and primers.

Video S1 iai.00355-23-s0003.mp4 Side-by-side swarming comparison of wild type, *serC*, and *guaA* mutants after incubation at 30C from 2-46 h.

ASM does not own the copyrights to Supplemental Material that may be linked to, or accessed through, an article. The authors have granted ASM a non-exclusive, world-wide license to publish the Supplemental Material files. Please contact the corresponding author directly for reuse.
