# Supplementary material for: Organ agar serves as physiologically relevant alternative for in vivo bacterial colonization
Source: Infect Immun. 2023 Oct 18;91(11):e00355-23. doi: 10.1128/iai.00355-23 (PMC10652904; doi:10.1128/iai.00355-23)
Supplement: Supplemental Figs S1-S8 — Supplemental figures. [file iai.00355-23-s0001.pdf]

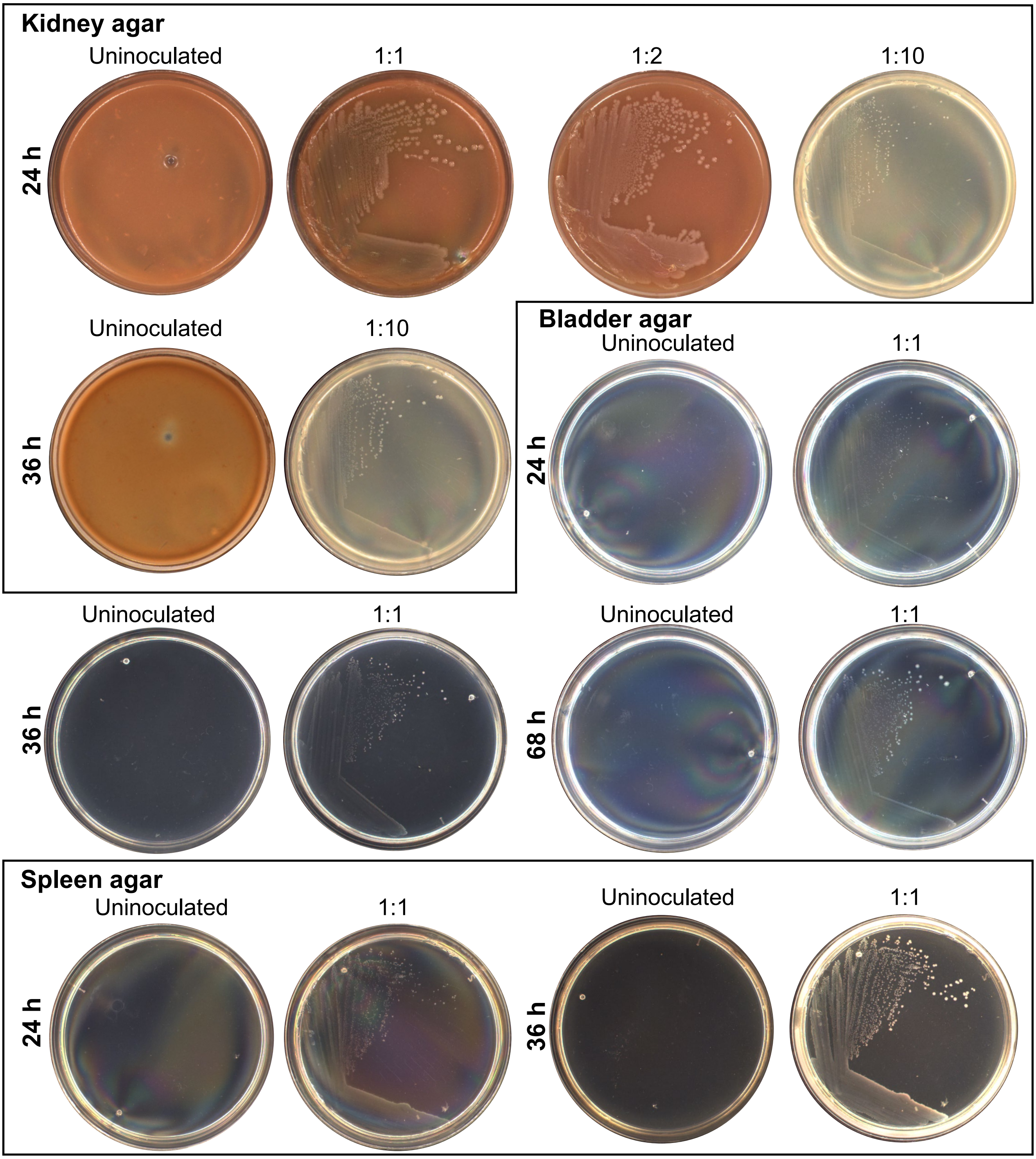

Supplemental Fig. S1. Optimizing organ agar. A single colony of *P. mirabilis* HI4320 was streaked on organ agars and incubated at 37°C for the indicated times. On kidney agar, colonies were visible within 24 h up to a 1:10 dilution, whereas colonies on bladder or spleen agar were only visible on undiluted agar, and became more visible following longer incubation. Thus, larger organs such as kidneys or livers facilitate larger screens with fewer mice.

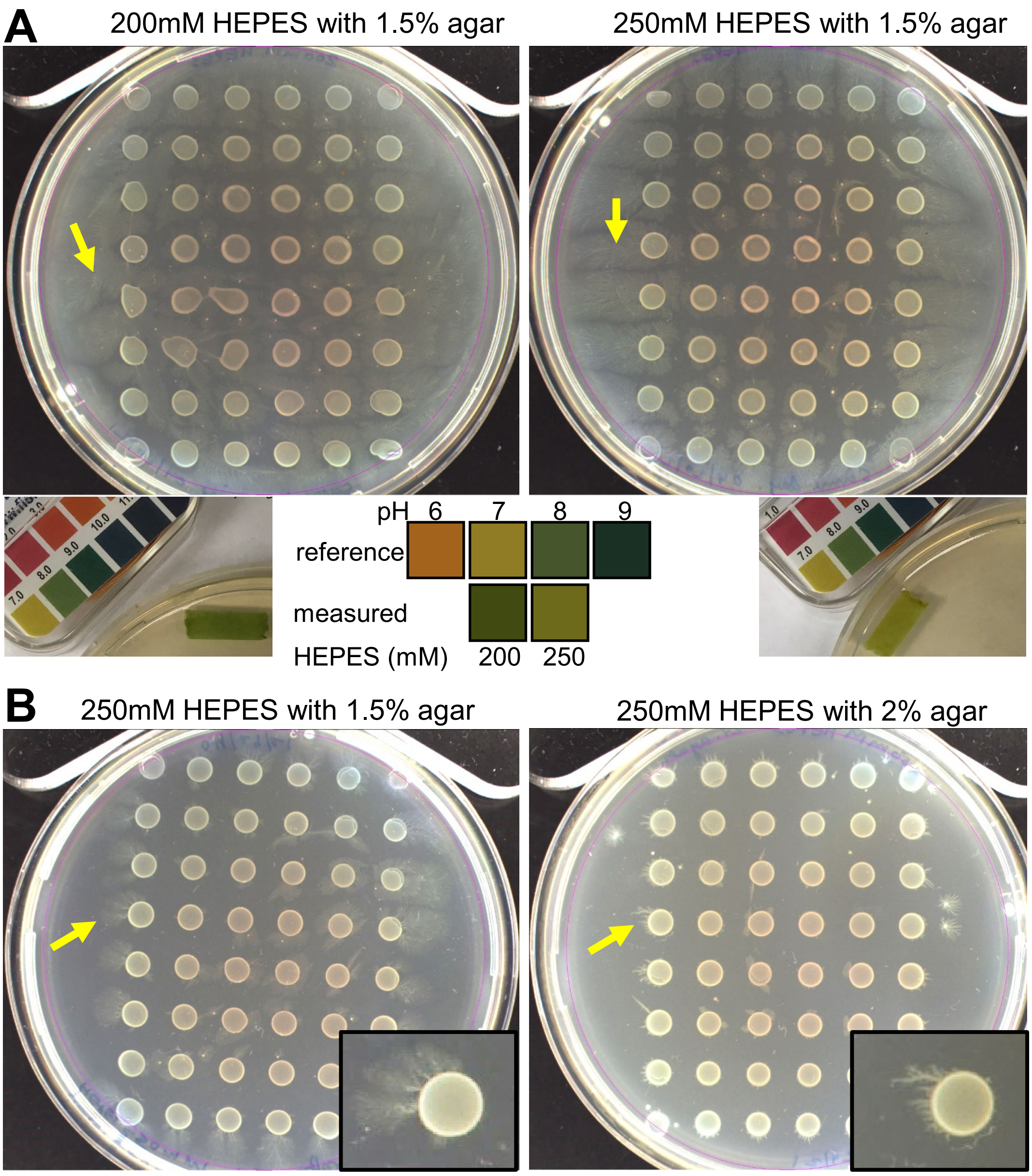

Supplemental Fig. S2. Optimizing urine agar. Wild-type *P. mirabilis* HI4320 was stamped onto agar made from human urine and incubated at 37°C for 24 h. **(A)** Comparison of buffering capacity. Urine agar was buffered with either 200 mM or 250 mM HEPES (pH 6.8), and pH paper placed on the agar surface after incubation was used to gauge the agar pH of each plate. 200 mM HEPES was insufficient to hold the pH below 8. Swarming motility from the points of inoculation is visible on both plates (arrows). **(B)** Comparison of agar concentration. Increasing the agar concentration from 1.5 to 2% greatly reduced swarming motility (arrows). Insets show close-ups of the arrow-indicated colonies, with increased contrast to make the swarms more visible.

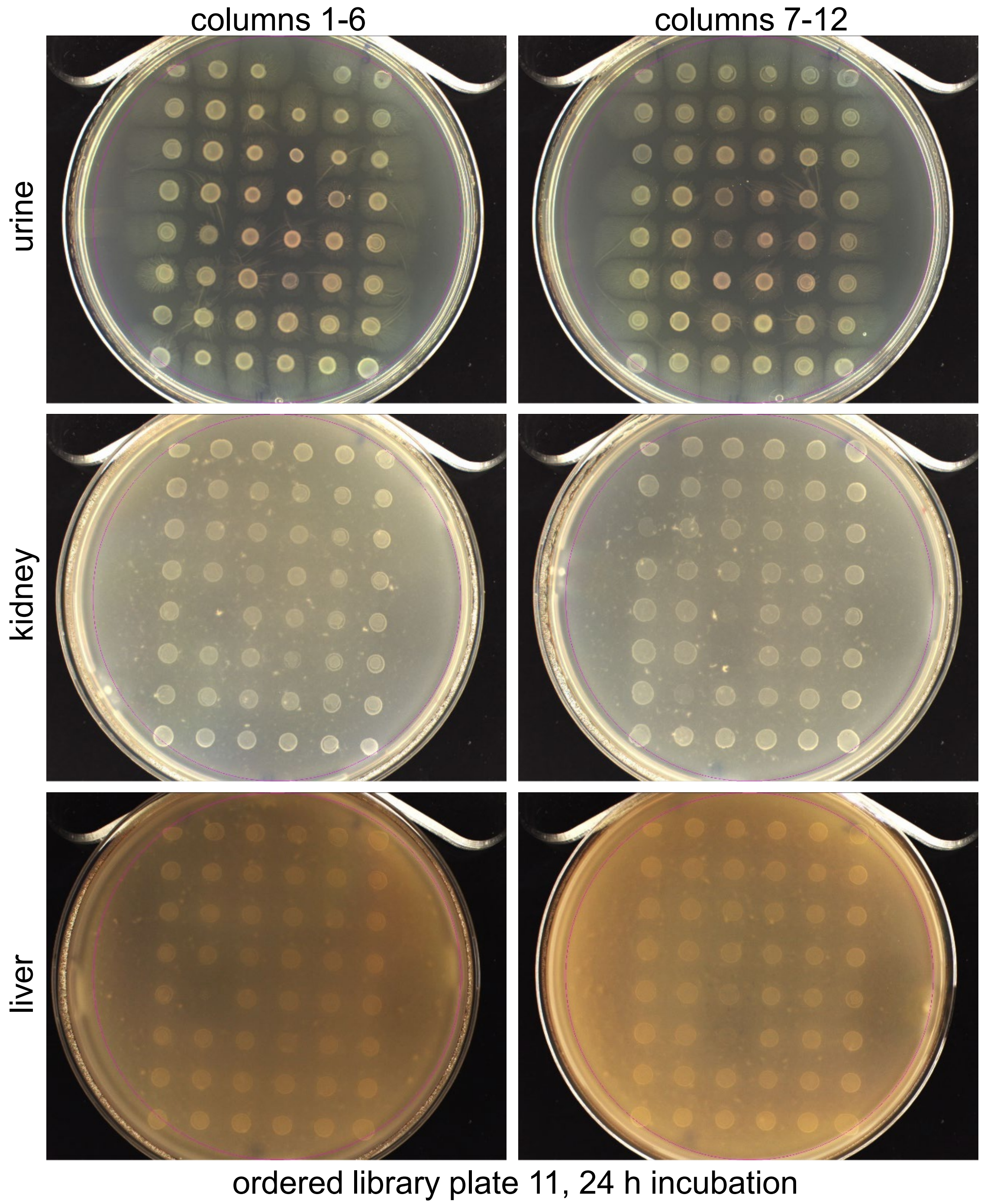

Supplemental Fig. S3. Example of organ agar screening from ordered transposon library. The screen of plate 11 from the ordered library is shown here at 24 hpi. Each Petri dish accommodates 48 samples; thus, two agar plates per organ were required to screen one 96-well plate of transposon mutants. Mutants with differential growth on urine, kidney, or liver agars can be seen.

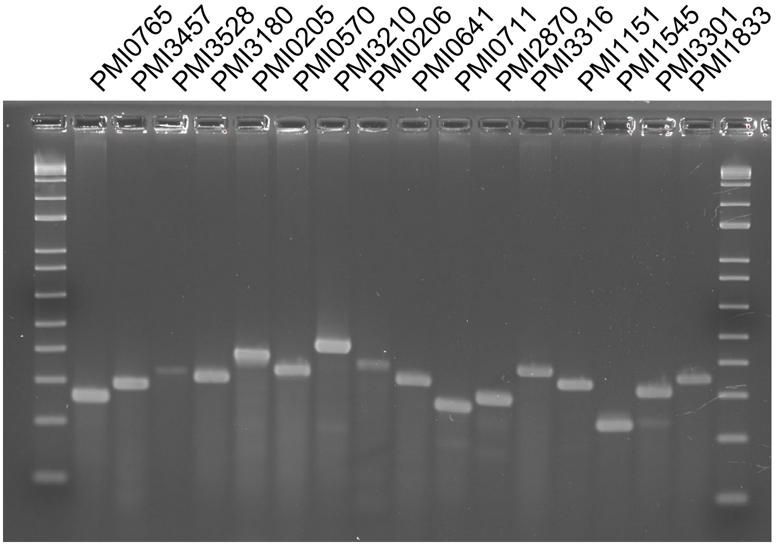

Supplemental Fig. S4. Confirmation of transposon insertions in ordered library. PCR using transposon-specific primer CP-7 and gene-specific primers was conducted to confirm the identify of transposon mutants in the *P. mirabilis* ordered library. The predicted insertion was confirmed for 16/20 mutants.

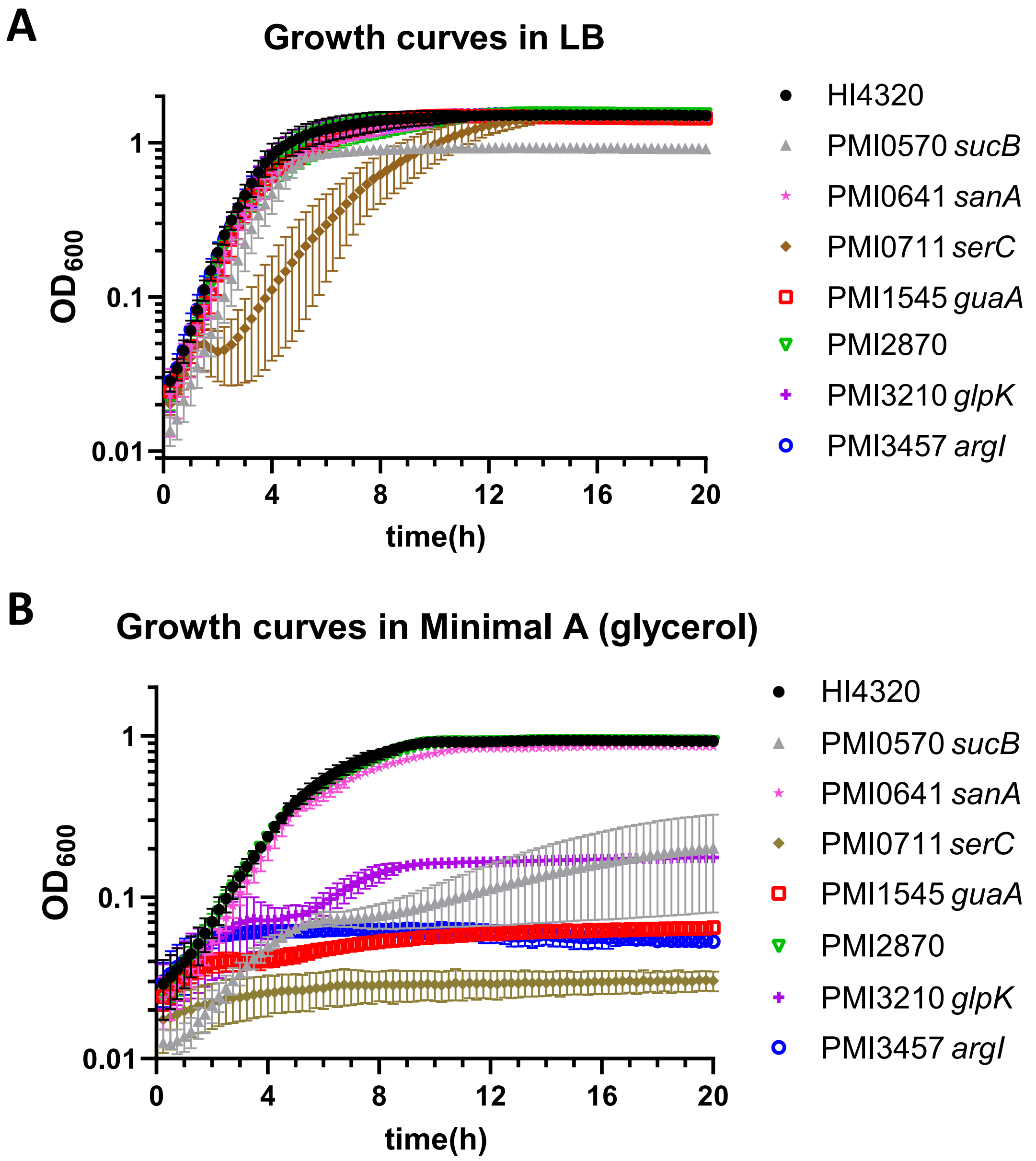

Supplemental Fig. S5. Growth curves for wild-type HI4320 and mutants. **(A)** LB; **(B)** Minimal A. These curves are the same data used to generate the area under the curve results shown in Fig. 3. Error bars show SD.

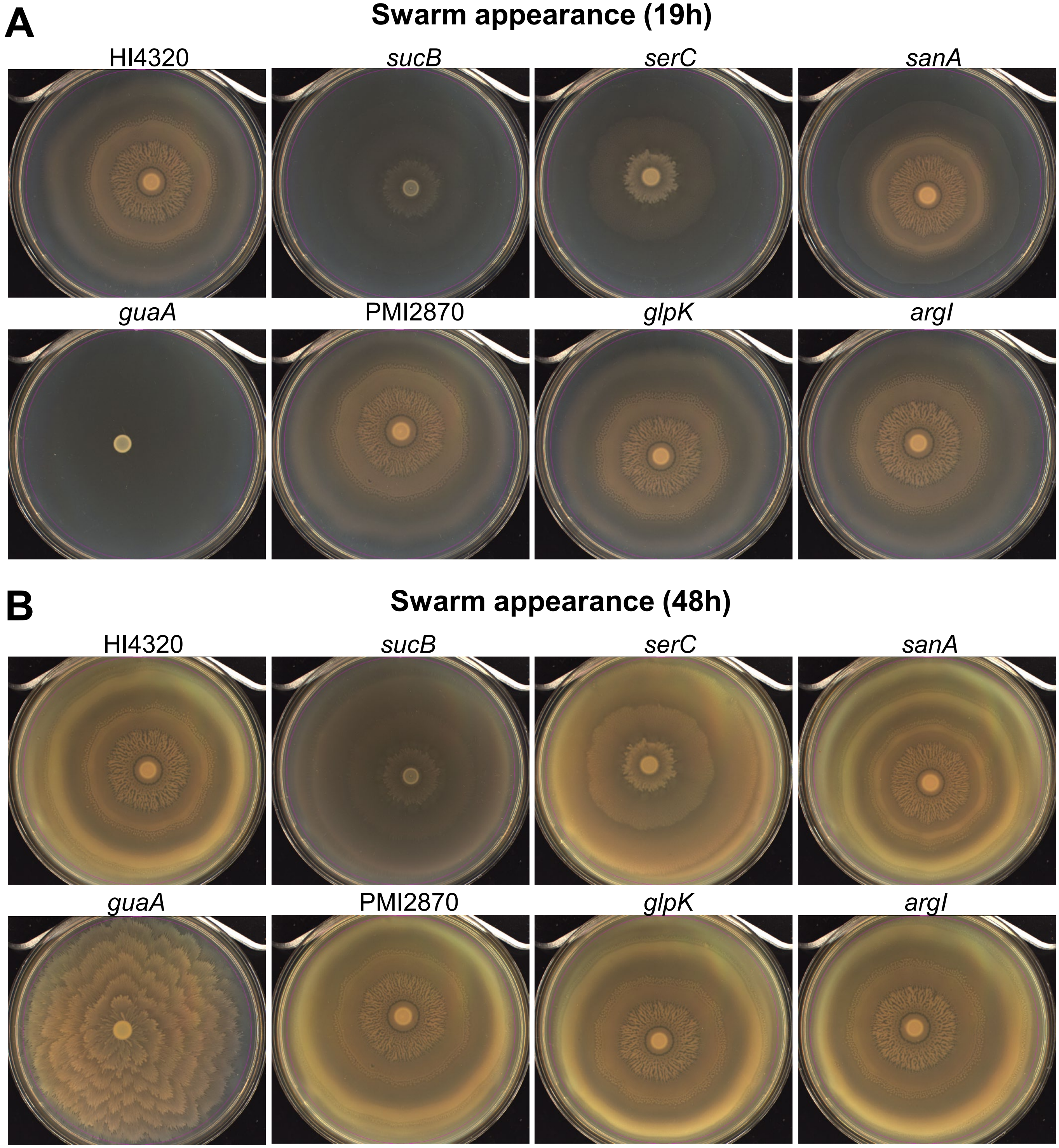

Supplemental Fig. S6. Swarming by *P. mirabilis* mutants. **(A)** Swarming after 19h at 30°C, before wild-type swarms reached the edge of the agar surface. **(B)** Swarming after 48h incubation at 30°C, when swarming and consolidation rings are more visible.

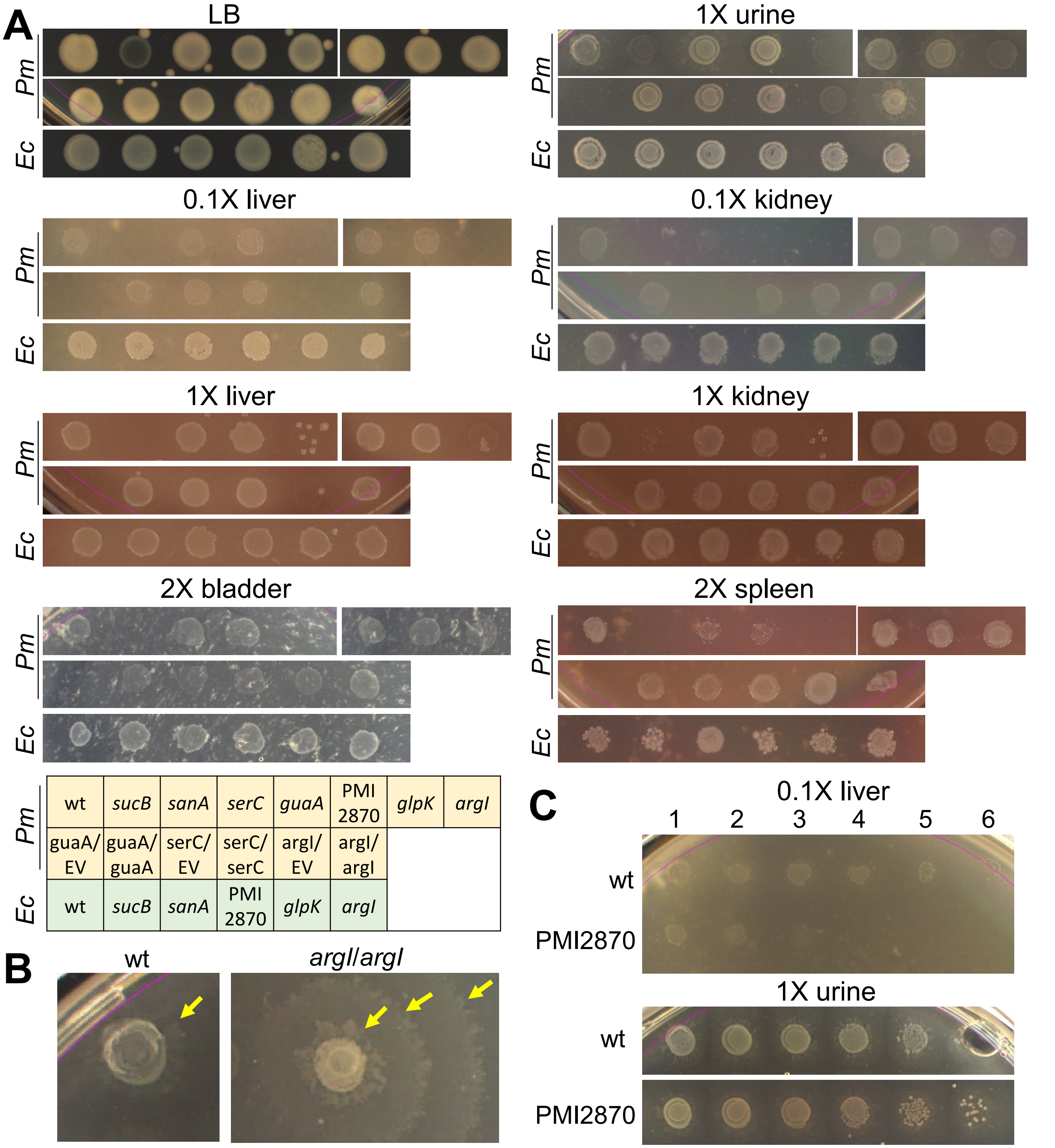

Supplemental Fig. S7. Organ agar made from UTI model mice gives similar results as agar made from outbred Swiss-Webster mice. (A) Targetron mutants were cultured in LB medium, adjusted to OD_{600} = 0.1, then stamped onto organ agar made from female CBA/J mice or agar made from pooled human urine. Photos were taken after 24 h incubation at 37°C. Homologous mutations in uropathogenic *E. coli* CFT073 pulled from an ordered transposon library were also tested, when available. Strains are indicated in the schematic at the bottom, with *P. mirabilis* (Pm) in yellow and *E. coli* (Ec) in green. **(B)** Magnification of wild-type *P. mirabilis* and the *argI* mutant complemented with *argI* on urine agar. Swarm rings are indicated with yellow arrows. **(C)** Dilution series on 1:10 liver agar showing growth defect for PMI2870 that is only visible when fewer bacteria were stamped. Column 1 was stamped from LB broth cultures that had been adjusted to OD_{600} = 1.0. Columns 2-6 are serial 1:10 dilutions. A similar experiment on urine agar did not reveal a defect for this mutant in a dilution series.

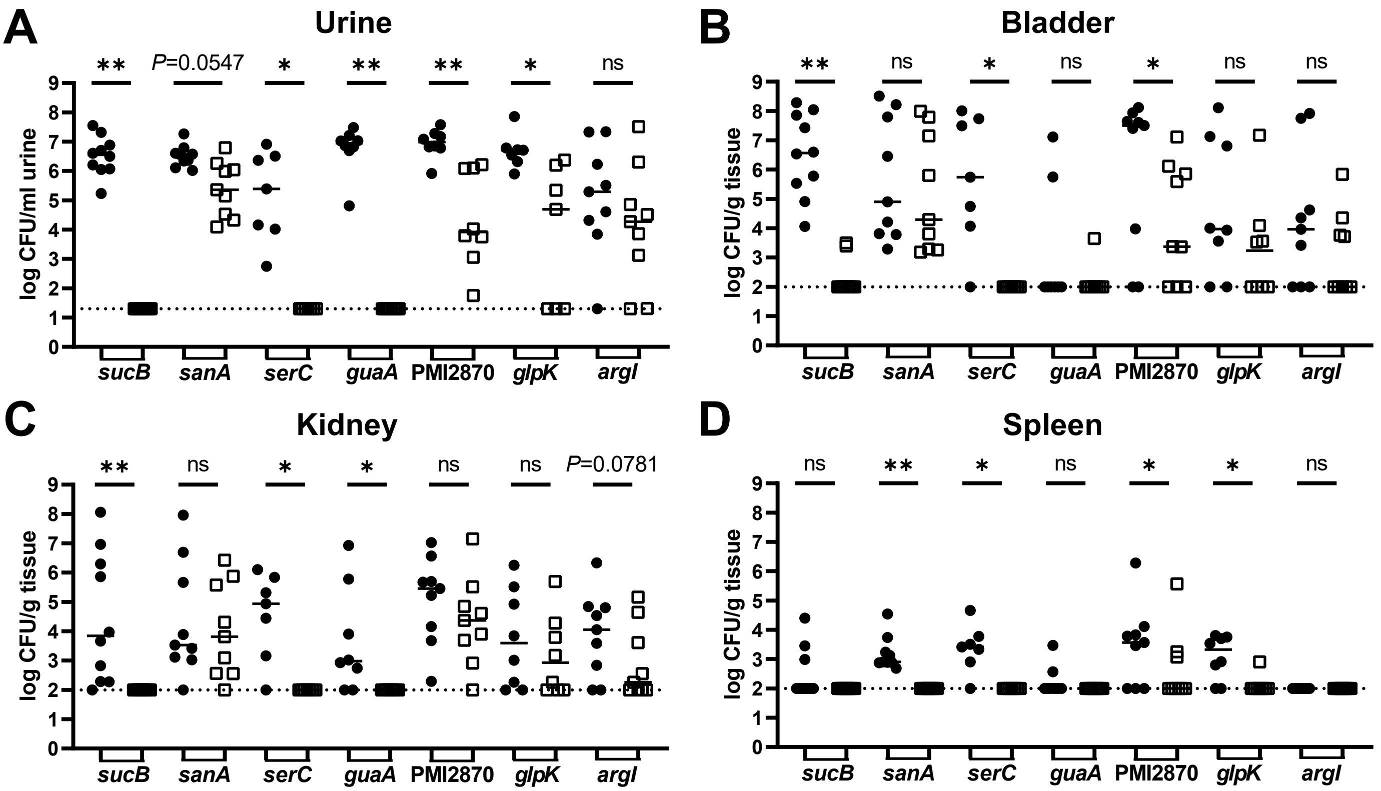

Supplemental Fig. S8. Total bacterial recovery from murine cochallenge experiment shown in Fig. 6. Female CBA/J mice were transurethrally inoculated with a 1:1 mixture of HI4320 (wt) and the indicated mutant. After 7 days, urine was collected, then organs were collected, homogenized, and plated to enumerate bacterial burden. Bars indicate median values. Dotted lines show the limit of detection (for urine, 20; for organ homogenates, 100). *P* values were assessed using the Wilcoxon signed rank test (**P*<0.05; ***P*<0.01; for *P* values between 0.1 and 0.05, the exact value is given; ns, not significant). ●, wt; □, mutant.
