# Supplementary material for: Organ agar serves as physiologically relevant alternative for in vivo bacterial colonization
Source: Infect Immun. 2023 Oct 18;91(11):e00355-23. doi: 10.1128/iai.00355-23 (PMC10652904; doi:10.1128/iai.00355-23)
Supplement: Supplemental Tables S1-S2 — Bacterial strains, plasmids, and primers. [file iai.00355-23-s0002.pdf]

Supplemental Table S1. Bacterial strains and plasmids

Bacterial strains used in this study

Name	Selection	Description	Reference
HI4320	tet	Wild-type clinical isolate of *P. mirabilis*	1
Top 10		Routine *E. coli* host for cloning	Invitrogen
PMI0570::kan	kan	*sucB* targetron mutant	This study
PMI0641::kan	kan	*sanA* targetron mutant	This study
PMI0711::kan	kan	*serC* targetron mutant	This study
PMI1545::kan	kan	*guaA* targetron mutant	This study
PMI2870::kan	kan	PMI2870 targetron mutant	This study
PMI3210::kan	kan	*glpK* targetron mutant	This study
PMI3457::kan	kan	*argI* targetron mutant	This study
CFT073		Wild-type clinical isolate of *E. coli*	2
CFT073 *sucB*::Tn	kan	*sucB* transposon mutant from ordered library	3
CFT073 *sanA*::Tn	kan	*sanA* transposon mutant from ordered library	3
CFT073 *yicH*::Tn	kan	*PMI2870* transposon mutant from ordered library	3
CFT073 *glpK*::Tn	kan	*glpK* transposon mutant from ordered library	3
CFT073 *argI*::Tn	kan	*argI* transposon mutant from ordered library	3

Plasmids used in this study

Name	Selection	Description	Reference
pAR1219	amp	T7 helper plasmid for targetron mutagenesis	4
pACD4K-CloxP-lacZ	chl	Targetron mutagenesis vector	Millipore Sigma
pSNS-2A1	chl	pACD4K-CloxP with intron targeting PMI3457 *argI*	This study
pSNS-2A2	chl	pACD4K-CloxP with intron targeting PMI0570 *sucB*	This study
pSNS-2A3	chl	pACD4K-CloxP with intron targeting PMI2870	This study
pSNS-2A4	chl	pACD4K-CloxP with intron targeting PMI3210 *glpK*	This study
pSNS-2A5	chl	pACD4K-CloxP with intron targeting PMI0641 *sanA*	This study
pSNS-2A7	chl	pACD4K-CloxP with intron targeting PMI0711 *serC*	This study
pSNS-2A8	chl	pACD4K-CloxP with intron targeting PMI1545 *guaA*	This study
pGEN-MCS	amp	Complementation plasmid; stably maintained without selection	5
guaA	amp	promoter	This study
pGEN-serC	amp	Complementation of *serC* under its native promoter	This study
pGEN-argI	amp	Complementation of *argI* under its native promoter	This study

References

1. Mobley HLT, Warren JW. Urease-positive bacteriuria and obstruction of long-term urinary catheters. J Clin Microbiol. 1987;25:2216-7.

2. Mobley HLT, Green DM, Trifillis AL, Johnson DE, Chippendale GR, Lockatell CV, Jones BD, Warren JW. Phelonephritogenic *Escherhichia coli* and killing of cultured human renal proximal tubular epithelia cells: role of hemolysin in some strains. Infect Immun. 1990;58:1281-9.

3. Shea AE, Marzoa J, Himpsl SD, Smith SN, Zhao L, Tran L, Mobley HLT. *Escherichia coli* CFT073 fitness factors during urinary tract infection: identification using an ordered transposon library. Appl Environ Microbiol. 2020;86(13). Epub 2020/05/03. doi: 10.1128/AEM.00691-20. PubMed PMID: 32358013; PMCID: PMC7301846.

4. Davanloo P, Rosenberg AH, Dunn JJ, Studier FW. Cloning and expression of the gene for bacteriophage T7 RNA polymerase. Proc Natl Acad Sci U S A. 1984;81(7):2035-9. Epub 1984/04/01. doi: 10.1073/pnas.81.7.2035. PubMed PMID: 6371808; PMCID: PMC345431.

5. Lane MC, Alteri CJ, Smith SN, Mobley HLT. Expression of flagella is coincident with uropathogenic *Escherichia coli* ascension to the upper urinary tract. Proc Natl Acad Sci U S A. 2007;104(42):16669-74.

Supplemental Table S2. Primers used in this study

Name	Sequence	Purpose
CP-7	CCAAGCAGAAGACGGCATACG	transposon outbound primer for confirmation of insertions
PMI0205 Rev	AGTTTGGTTGTCCCATCGTC	PCR confirmation of transposon insertion in PMI0205, paired with CP-7
PMI0206 Rev	AGATGTGGGGATTGCTAACG	PCR confirmation of transposon insertion in PMI0206, paired with CP-7
PMI0570 Rev	CGTCGTATCTTGTGCAGGTT	PCR confirmation of transposon insertion in PMI0570, paired with CP-7
PMI0641 Rev	TCCATAACGATACGCGATGC	PCR confirmation of transposon insertion in PMI0641, paired with CP-7
PMI0711 Rev	GCCACCATGACAGAACAGAA	PCR confirmation of transposon insertion in PMI0711, paired with CP-7
PMI0765 Rev	TCAAAACGACCGAAACCAGT	PCR confirmation of transposon insertion in PMI0765, paired with CP-7
PMI1151 Fwd	CCGCAATAAACCCTATCGGC	PCR confirmation of transposon insertion in PMI1151, paired with CP-7
PMI1545 Fwd	GCCGGTATTAGGCATCTGCT	PCR confirmation of transposon insertion in PMI1545, paired with CP-7
PMI1833 Fwd	ATTGCATGGCAGACCGAAGT	PCR confirmation of transposon insertion in PMI1833, paired with CP-7
PMI2870 Rev	GTCTTTGTCTCGCTGTTCCA	PCR confirmation of transposon insertion in PMI2870, paired with CP-7
PMI3180 Rev	TTTGTAAGCGGTTTTGCGTC	PCR confirmation of transposon insertion in PMI3180, paired with CP-7
PMI3210 Rev	GGCACCACTTTAGGTAGCAT	PCR confirmation of transposon insertion in PMI3210, paired with CP-7
PMI3301 Fwd	GTGAGCAGTCAACGACCTGT	PCR confirmation of transposon insertion in PMI3301, paired with CP-7
PMI3316 Rev	CACCAAACGAAACGCAAGAA	PCR confirmation of transposon insertion in PMI3316, paired with CP-7
PMI3457 Rev	AAGGATTTGCTAAGGTCGCC	PCR confirmation of transposon insertion in PMI3457, paired with CP-7
PMI3528 Rev	GGTAGATGCTGGAACCCTTG	PCR confirmation of transposon insertion in PMI3528, paired with CP-7
PMI0006 Fwd	AGCAGGTTGTGATCGCCTAA	PCR-confirmed lack of transposon insertion in PMI0006
PMI0006 Rev	ATCGCAGGTAAAGAGAAGGC	PCR-confirmed lack of transposon insertion in PMI0006
PMI3384 Fwd	CAAGACGGGAGGTTTACGCT	PCR-confirmed lack of transposon insertion in PMI3384
PMI3384 Rev	ACCACGCTGTACTGAGCTAT	PCR-confirmed lack of transposon insertion in PMI3384
PMI3431 Fwd	CGCGGAAAAGAACGGCTAATG	PCR-confirmed lack of transposon insertion in PMI3431
PMI3431 Rev	GAGGGAATAGCGGTGTAAGC	PCR-confirmed lack of transposon insertion in PMI3431
PMI3538 Fwd	CCATTTTTACCCGATGGGCG	PCR-confirmed lack of transposon insertion in PMI3538
PMI3538 Rev	TCAACGTGTAACTCAGCCAC	PCR-confirmed lack of transposon insertion in PMI3538
pACD4K-C-Gib-fwd	TAGGAGAACCTATGGGAAC	Amplification of targetron plasmid pACD4K-CloxP for Gibson cloning
pACD4K-C-Gib-rev	CTAGAGGGGAATTGTTATC	Amplification of targetron plasmid pACD4K-CloxP for Gibson cloning
intron-Gib-fwd	ggataacaattcccctctagAAAAAAGCTTATAATTATCCTTA	Amplification of targetron synthetic intron fragment for Gibson cloning
intron-Gib-rev	cgttcccataggttctcctaCAGATTGTACAAATGTGGTG	Amplification of targetron synthetic intron fragment for Gibson cloning
PMI0570-TTF	TTGTGTTAGAAGTACCGGCG	PCR confirmation of targetron insertion in PMI0570 *sucB*
PMI0570-TTR	GTCGGGCTAAGAGCATCATT	PCR confirmation of targetron insertion in PMI0570 *sucB*
PMI0641-TTF	GTTTGGGACAGATGGCTTTAC	PCR confirmation of targetron insertion in PMI0641 *sanA*
PMI0641-TTR	CTAGAAAACGCGGTTCACG	PCR confirmation of targetron insertion in PMI0641 *sanA*
PMI0711-TTF	TAGGTGAGAAAACCACCGC	PCR confirmation of targetron insertion in PMI0711 *serC*

PMI0711-TTR	AGCAATCCCATCGATAGTCTC	PCR confirmation of targetron insertion in PMI0711 *serC*
PMI1545-TTF	CGAGAAAGGCTTGAAAACAGA	PCR confirmation of targetron insertion in PMI1545 *guaA*
PMI1545-TTR	TCTTCGGTAACATCCCATGC	PCR confirmation of targetron insertion in PMI1545 *guaA*
PMI2870-TTF	CAATGGGGTGCGCAAAAA	PCR confirmation of targetron insertion in PMI2870
PMI2870-TTR	CAGATGAGACGCCGTAGTAA	PCR confirmation of targetron insertion in PMI2870
PMI3210-TTF	TGAAACGACTATCGTCTGGG	PCR confirmation of targetron insertion in PMI3210 *glpK*
PMI3210-TTR	CGCCTTCAACATTATCGAGG	PCR confirmation of targetron insertion in PMI3210 *glpK*
PMI3457-TTF	AAAAGATTCCACCCGTACCC	PCR confirmation of targetron insertion in PMI3457 *argI*
PMI3457-TTR	TCAGCATATTTTGCCAGCAT	PCR confirmation of targetron insertion in PMI3457 *argI*
pGEN-GibsonF	AAGATCCCAACGAAAAGC	Amplification of complementation plasmid pGEN-MCS for Gibson cloning
pGEN-GibsonR	CAAGCTTCATATGCCCGG	Amplification of complementation plasmid pGEN-MCS for Gibson cloning
PguaBA-GibF	ccccgggcatatgaagcttgATCAATAAAAATTTTCAAAAAAAGCTAG	Amplification of *guaBA* promoter for Gibson cloning
PguaBA-GibR	ttgctgtcatagcaatatctcaccaagg	Amplification of *guaBA* promoter for Gibson cloning
guaA-GibF	agatattgctatgacagcaaatatccataatc	Amplification of *guaA* gene for Gibson cloning
guaA-GibR	acgcttttcgttgggatcttttaTTCCCACTCAATCGTTG	Amplification of *guaA* gene for Gibson cloning
serC_GibF	ccccgggcatatgaagcttgGTTTACAAAAGTCATTGCGCCAATTAG	Amplification of *serC* and promoter for Gibson cloning
serC_GibR	acgcttttcgttgggatcttttaAGCGTGGCGACGCTC	Amplification of *serC* and promoter for Gibson cloning
argI_GibF	ccccgggcatatgaagcttgCAAAAGTGACGTTTAAGG	Amplification of *argI* and promoter for Gibson cloning
argI_GibR	acgcttttcgttgggatcttttaAAAAGGATTTGCTAAGGTC	Amplification of *argI* and promoter for Gibson cloning
